# Same-day HIV testing with initiation of antiretroviral therapy versus standard care for persons living with HIV: A randomized unblinded trial

**DOI:** 10.1371/journal.pmed.1002357

**Published:** 2017-07-25

**Authors:** Serena P. Koenig, Nancy Dorvil, Jessy G. Dévieux, Bethany L. Hedt-Gauthier, Cynthia Riviere, Mikerlyne Faustin, Kerlyne Lavoile, Christian Perodin, Alexandra Apollon, Limathe Duverger, Margaret L. McNairy, Kelly A. Hennessey, Ariadne Souroutzidis, Pierre-Yves Cremieux, Patrice Severe, Jean W. Pape

**Affiliations:** 1 Haitian Study Group for Kaposi’s Sarcoma and Opportunistic Infections (GHESKIO), Port-au-Prince, Haiti; 2 Division of Global Health Equity, Brigham and Women’s Hospital, Boston, Massachusetts, United States of America; 3 AIDS Prevention Program, Florida International University, Miami, Florida, United States of America; 4 Department of Global Health and Social Medicine, Harvard Medical School, Harvard University, Boston, Massachusetts, United States of America; 5 Center for Global Health, Department of Medicine, Weill Cornell Medical College, Cornell University, New York, New York, United States of America; 6 Division of General Internal Medicine, Department of Medicine, Weill Cornell Medical College, Cornell University, New York, New York, United States of America; 7 Analysis Group, Boston, Massachusetts, United States of America; University of California, San Francisco, UNITED STATES

## Abstract

**Background:**

Attrition during the period from HIV testing to antiretroviral therapy (ART) initiation is high worldwide. We assessed whether same-day HIV testing and ART initiation improves retention and virologic suppression.

**Methods and findings:**

We conducted an unblinded, randomized trial of standard ART initiation versus same-day HIV testing and ART initiation among eligible adults ≥18 years old with World Health Organization Stage 1 or 2 disease and CD4 count ≤500 cells/mm^3^. The study was conducted among outpatients at the Haitian Group for the Study of Kaposi’s Sarcoma and Opportunistic infections (GHESKIO) Clinic in Port-au-Prince, Haiti. Participants were randomly assigned (1:1) to standard ART initiation or same-day HIV testing and ART initiation. The standard group initiated ART 3 weeks after HIV testing, and the same-day group initiated ART on the day of testing. The primary study endpoint was retention in care 12 months after HIV testing with HIV-1 RNA <50 copies/ml. We assessed the impact of treatment arm with a modified intention-to-treat analysis, using multivariable logistic regression controlling for potential confounders. Between August 2013 and October 2015, 762 participants were enrolled; 59 participants transferred to other clinics during the study period, and were excluded as per protocol, leaving 356 in the standard and 347 in the same-day ART groups. In the standard ART group, 156 (44%) participants were retained in care with 12-month HIV-1 RNA <50 copies, and 184 (52%) had <1,000 copies/ml; 20 participants (6%) died. In the same-day ART group, 184 (53%) participants were retained with HIV-1 RNA <50 copies/ml, and 212 (61%) had <1,000 copies/ml; 10 (3%) participants died. The unadjusted risk ratio (RR) of being retained at 12 months with HIV-1 RNA <50 copies/ml was 1.21 (95% CI: 1.04, 1.38; *p* = 0.015) for the same-day ART group compared to the standard ART group, and the unadjusted RR for being retained with HIV-1 RNA <1,000 copies was 1.18 (95% CI: 1.04, 1.31; *p* = 0.012). The main limitation of this study is that it was conducted at a single urban clinic, and the generalizability to other settings is uncertain.

**Conclusions:**

Same-day HIV testing and ART initiation is feasible and beneficial in this setting, as it improves retention in care with virologic suppression among patients with early clinical HIV disease.

**Trial registration:**

This study is registered with ClinicalTrials.gov number NCT01900080

## Abstract 2

In a randomized unblinded trial in Port-au-Prince, Haiti, Serena Koenig and colleagues investigate whether initiating ART on the day of HIV diagnosis improved retention in care and viral suppression.

## Author summary

**Why was this study done?:**

- Multiple visits for counseling, laboratory testing, and other procedures to prepare patients for initiation of antiretroviral therapy (ART) are burdensome and contribute to the high rate of attrition during the period from HIV testing to ART initiation.
- The World Health Organization (WHO) recently changed their guidelines to recommend ART for all persons living with HIV, facilitating ART initiation.
- This study was conducted to determine if ART initiation on the day of HIV diagnosis could improve treatment initiation rates, retention in care, and HIV viral suppression for patients with asymptomatic or minimally symptomatic HIV disease.

**What did the researchers do and find?:**

- We randomly assigned patients who presented for HIV testing at a clinic in Port-au-Prince, Haiti to standard ART initiation or same-day HIV testing and ART initiation (356 in the standard and 347 in the same-day groups).
- The standard group had 3 weekly visits with a social worker and physician and then started ART 21 days after the date of HIV diagnosis; the same-day ART group initiated ART on the day of HIV diagnosis.
- All participants in the same-day ART group and 92% of participants in the standard group initiated ART.
- At 12 months after HIV testing, a higher proportion of participants in the same-day ART group were retained in care (80% versus 72%), and a higher proportion were retained in care with viral load <50 copies/ml (53% versus 44%) and viral load <1,000 copies/ml (61% versus 52%).

**What do these findings mean?:**

- This study demonstrates that it is feasible to initiate ART on the day of HIV diagnosis for patients with early HIV clinical disease and that same-day treatment leads to increased ART uptake, retention in care, and viral suppression.
- Though same-day ART initiation improves outcomes, retention in care and viral suppression remain suboptimal, so further interventions to maximize long-term outcomes will be essential.
- The study is limited by being conducted at 1 clinic in urban Haiti. Further study will be necessary to determine if this strategy will be effective in other settings.

## Introduction

The Joint United Nations Programme on HIV/AIDS (UNAIDS) 90-90-90 targets state that 90% of HIV-infected persons know their status, 90% initiate antiretroviral therapy (ART), and 90% achieve virologic suppression by the year 2020 to curb the AIDS epidemic [1]. In 2015, the World Health Organization (WHO) updated their guidelines to recommend ART for all persons living with HIV based on evidence that earlier treatment improves outcomes and decreases transmission [2–4]. To achieve these goals, patients must be promptly linked to HIV services, initiated on ART, and retained in lifelong care [5].

Attrition rates are particularly high during the period from HIV testing to ART initiation, with one-quarter to one-third of patients lost in the process of starting ART [6–9]. Even if many of these patients re-engage in care at a later date, they will return with more advanced disease. Though there are many factors that contribute to pretreatment attrition, the current standard of care in most settings, which requires multiple sequential visits for HIV testing and counseling, laboratory testing, and adherence counseling prior to ART initiation, creates barriers to treatment initiation. As of June 2016, WHO guidelines note inadequate evidence to support a recommendation of same-day HIV testing and ART initiation [2]. However, the availability of point-of-care tests, the fact that CD4 cell counts are no longer necessary prior to ART initiation, and the provision of same-day counseling can accelerate treatment initiation, potentially reducing attrition [10–12]. We conducted a randomized trial in Haiti to determine whether same-day HIV testing and ART initiation, as compared with standard ART initiation, improves retention in care with viral suppression.

## Methods

### Study design and setting

We conducted an unblinded, randomized controlled trial of standard ART initiation versus same-day HIV testing and ART initiation among HIV-infected adults at the Haitian Group for the Study of Kaposi’s Sarcoma and Opportunistic infections (GHESKIO) in Port-au-Prince, Haiti. Haiti is the poorest country in the Western Hemisphere, with adult HIV prevalence of 1.7% [13,14]. GHESKIO is a Haitian nongovernmental organization and the largest provider of HIV care in the Caribbean, treating up to 700 patients per day for HIV and/or tuberculosis (TB). All care is provided free of charge. The study was approved by the institutional review boards at Partners Healthcare, GHESKIO, Weill Cornell Medical College, and Florida International University. See supporting information files S1 Text for the study protocol and S2 Text for the CONSORT checklist.

### Participants

Participants were recruited from the HIV voluntary counseling and testing center at GHESKIO from August 2013 to October 2015. They received HIV testing and posttest counseling; those with a positive HIV test were referred for same-day physician evaluation, CD4 count (FACS Count, Becton-Dickinson, Franklin Lakes, New Jersey), WHO staging, and chest radiograph. Patients were eligible for study inclusion if they were infected with HIV-1, ≥18 years of age, and had WHO Stage 1 or 2 disease and CD4 count ≤500 cells/mm^3^. Initially, enrollment was limited to patients with CD4 count ≤350 cells/mm^3^, but in February 2014, the cutoff was increased to ≤500 cells/mm^3^ in response to revised WHO and Haitian guidelines [15]. Patients were excluded if they were already aware of their HIV diagnosis, had received ART previously, were pregnant or breastfeeding, lived outside of the greater Port-au-Prince metropolitan area, planned to transfer care during the study period, or failed to demonstrate preparedness on an ART readiness survey, which was administered by a social worker prior to study enrollment. The survey includes a 5-point scale, with respondents ranking their preparedness from “not at all ready” to “completely ready” in response to 7 questions. Study inclusion required a response of “somewhat ready” or “completely ready” for all 7 questions (S3 Text) [16].

### Randomization and masking

After the patients had provided written informed consent, the study team performed a screening evaluation for study exclusion criteria, and eligible participants were enrolled and randomized on the day of HIV testing. Participants were randomly assigned with the use of a computer-generated random-number list to either standard ART or same-day ART initiation in a 1:1 ratio, with allocation concealment. The randomization sequence was generated by a computer in the GHESKIO data management unit by a data manager who had no other involvement in study procedures. Participants were enrolled in the study and assigned to groups by a study physician. Participants, site personnel, and study statisticians were not masked to group assignment.

### Procedures

**Fig 1:**
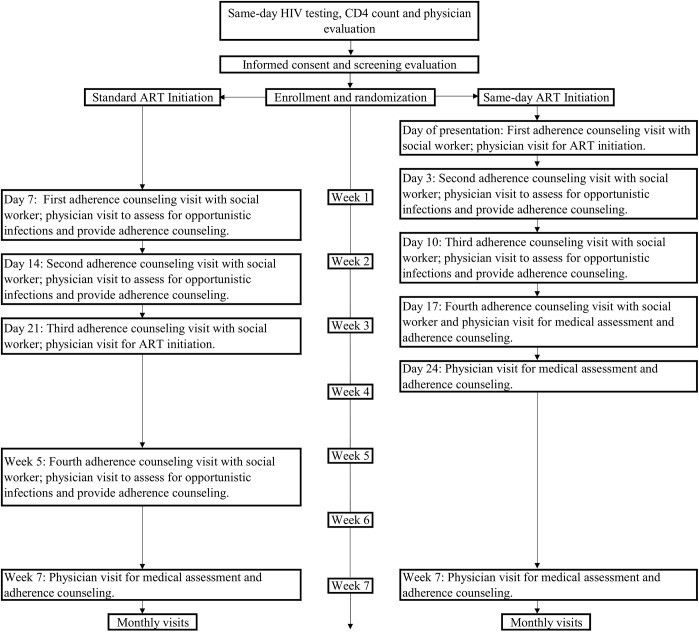
Study interventions for the standard ART and same-day ART groups.

After randomization, the standard group participants received ART initiation procedures that mirror national guidelines. Participants were referred to return on Day 7 for baseline laboratory tests (creatinine, alanine aminotransferase, aspartate aminotransferase, complete blood count, purified protein derivative [PPD]), physician evaluation, and counseling with a social worker. On Day 10, they received interpretation of PPD results, and on Days 14 and 21, they were seen by a physician and social worker for additional counseling, test results, and ongoing evaluations for opportunistic infections. Participants started ART on Day 21 and had an additional social worker and physician visit at Week 5 (Fig 1). The ART regimen was the same as that for nonstudy patients at GHESKIO. First-line therapy included a single combination tablet including tenofovir disoproxil fumarate, lamivudine, and efavirenz.

The same-day ART group had identical laboratory tests as the standard ART group, a 30-minute counseling session with a social worker, and physician evaluation, and then initiated the same ART regimen as the standard ART group. They returned on Day 3 for physician and social worker visits and receipt of baseline laboratory test results; those with creatinine clearance <50 mL/minute as calculated by the Cockcroft-Gault equation were switched from tenofovir to zidovudine or abacavir. They returned on Days 10 and 17 for additional physician and social worker visits and on Day 24 for a physician visit. The same number of scheduled physician visits and counseling sessions were provided to each group so that the only difference in care was in the schedule of visits during the first 5 weeks of the study and the timing of ART initiation.

All care was delivered by GHESKIO clinic staff, and the same providers (physicians, nurses, social workers, pharmacists, and field workers) cared for both groups. A counseling manual was followed with an outline for the social workers to follow at each scheduled counseling visit; these were identical between groups, except for the timing of ART initiation, and each session took about 30 minutes. All counseling was provided for individual patients, rather than for groups. The counseling sessions were audiotaped and systematically evaluated for quality control purposes. If a participant in either group missed a study visit that included a scheduled social worker counseling session, the counseling was provided at the next visit.

Participants in both groups had monthly physician visits throughout the follow-up period and received the same package of services provided to all HIV-infected patients at GHESKIO, including prophylactic treatment with trimethoprim-sulfamethoxazole and isoniazid. Field workers phoned patients who missed a visit and attempted a home visit for those not reachable by phone. Participants received a transportation subsidy of 100 Haitian gourdes (US$1.70) per visit.

### Outcomes

The primary endpoint was retention in care with HIV-1 RNA <50 copies/ml at 12 months after HIV testing. Retention was defined as attending the 12-month visit (1 clinic visit between 12 and 15 months after HIV testing). Lost to follow-up (LTFU) was defined as failure to attend the 12-month visit. Deaths were ascertained by review of medical records or report from family members. A National Institutes of Health Division of AIDS Expedited Adverse Event Form was filled out within 48 hours after the study team became aware of any death. Transfers were ascertained by confirmation that the participant was receiving care at a different site. Secondary outcomes include survival, ART initiation, retention in care with HIV-1 RNA <1,000 copies/ml at 12 months after HIV testing, adherence as measured by pharmacy refill records and self-report, and cost and cost-effectiveness of standard and same-day ART; the adherence and cost-effectiveness evaluations will be reported in separate manuscripts.

### Statistical analysis

Demographic, clinical, and laboratory data from the electronic medical record and study forms were de-identified, entered into an Excel spreadsheet, and exported into Stata v14 software (StataCorp, 2011, College Station, Texas) for analysis. After study completion, all participants who were LTFU were recontacted to determine their vital status.

The study was powered to detect a 10% absolute difference in the rate of retention with virologic suppression between the 2 groups at 12 months after enrollment (65% in the standard and 75% in the same-day ART group). At the α = 0.05 significance level, we estimated that we would need to enroll 349 participants per group (698 in total) to achieve 80% power to detect this difference. Because patients who transferred during the study period were excluded, we increased the total sample size to 762 participants. For all analyses, a modified intention-to-treat approach was used, in which all patients were analyzed according to their assignment group, excluding patients who transferred to another facility during the follow-up period, according to protocol.

Baseline characteristics were summarized using simple frequencies and proportions and medians with interquartile ranges (IQRs) stratified by treatment arm. Among participants who died, baseline CD4 count was compared using the Wilcoxon rank-sum test. We compared the proportion of participants who were retained in care with HIV-1 RNA <50 copies/ml (primary endpoint), retained with HIV-1 RNA <1,000 copies/ml, retained regardless of HIV-1 RNA, initiated ART, and died (secondary endpoints) at 12 months after enrollment using a chi-square test. We conducted multivariable logistic regression including all covariates listed in Table 1 to control for any residual confounding. We present unadjusted and adjusted risk ratios (RR) with 95% confidence intervals. Because of the change in enrollment criteria mid-study, we conducted a sensitivity analysis that included only the participants who met the original enrollment criteria of CD4 count ≤350 cells/mm^3^. In response to a reviewer’s request, we also plotted retention in care, regardless of viral load, for both groups and compared the distributions with the log-rank test. The study is registered with ClinicalTrials.gov number NCT01900080.

**Table 1:** Baseline characteristics of study participants by group.

Characteristic	Standard Group (*n* = 356)	Same-Day ART Group (*n* = 347)
**Age (years)—Median (IQR)**	37 (30, 45)	37 (29, 46)
**Female sex—no. (%)**	181 (51)	166 (48)
**Education—no. (%)**		
No school	90 (25)	93 (27)
Primary school	110 (31)	111 (32)
Secondary school or more	156 (44)	143 (41)
**Income—no. (%)**		
No income	92 (26)	90 (26)
>$0 to $1/day	176 (49)	159 (46)
>$1 to $2/day	67 (19)	76 (22)
>$2/day	21 (6)	22 (6)
**Marital status—no. (%)**		
Single	71 (20)	71 (20)
Currently married/living with partner	222 (62)	211 (61)
Formerly married	63 (18)	65 (19)
**WHO Stage—no. (%)**		
WHO Stage 1	117 (33)	101 (29)
WHO Stage 2	239 (67)	246 (71)
**CD4 count (cells/mm^3^)—Median (IQR)**	247 (150, 349)	249 (143, 336)
Body mass index—Median (IQR)*	21.6 (19.7, 23.9)	20.9 (19.3, 23.5)

* Body mass index differed significantly between the 2 groups (*p* = 0.025).

ART, antiretroviral therapy; IQR, interquartile range, WHO, World Health Organization.

## Results

**Fig 2:**
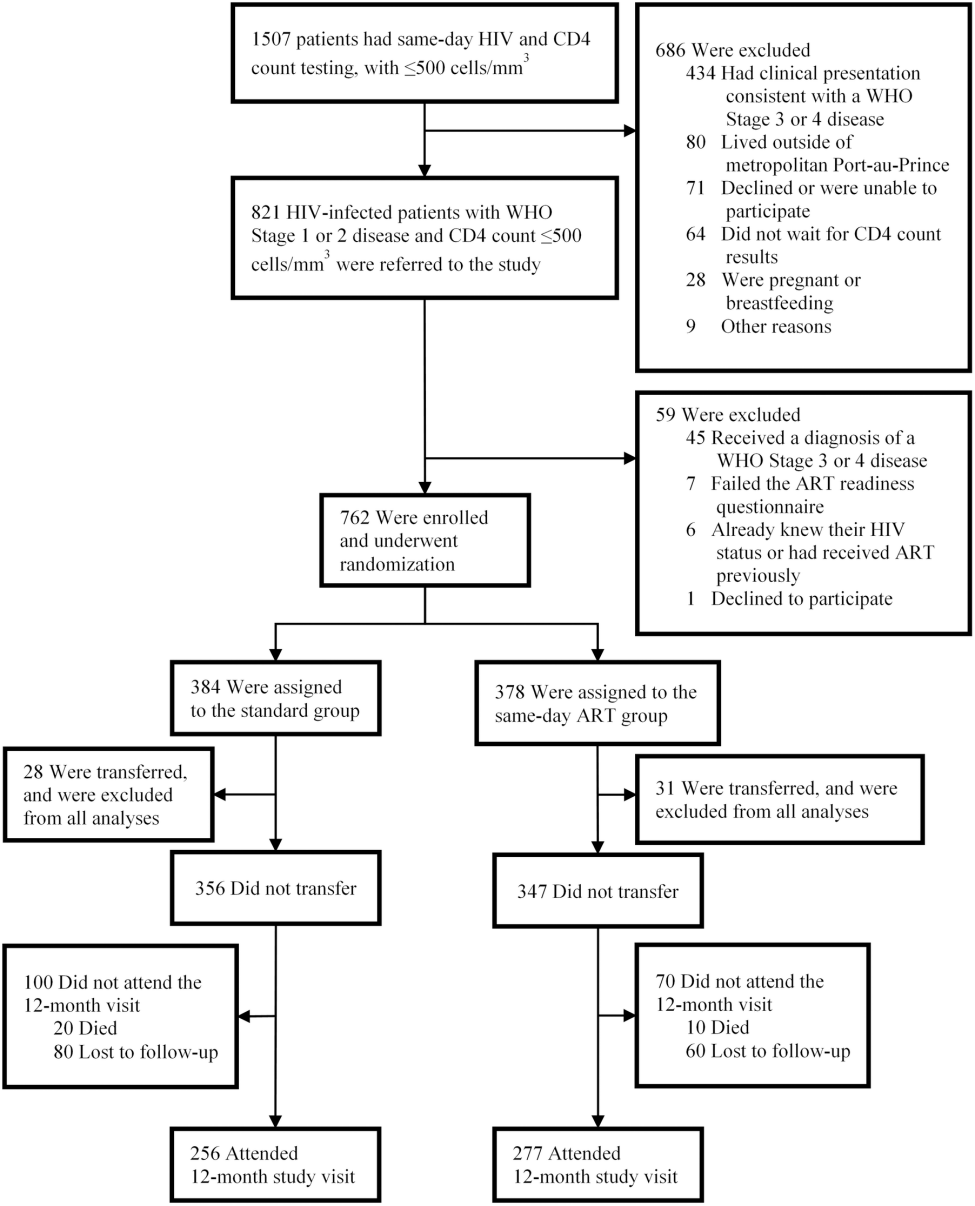
Screening, randomization, and follow-up.

A total of 821 patients were screened, and 762 were enrolled in the study and underwent randomization (Fig 2). After randomization, 59 participants (28 in the standard ART and 31 in same-day ART group) transferred to another clinic and were excluded from all analyses, as per protocol. The median age was 37 years old (IQR: 30–45 years), 347 (49%) were women, and the median CD4 count was 248 cells/mm^3^ (IQR: 148, 345).

Of the 356 participants in the standard group, 256 (72%) were retained in care, 20 (6%) died, and 80 (23%) were LTFU (Table 2). Among the 256 participants retained in the standard ART group, 156 (61% of retained and 44% overall) had HIV-1 RNA <50 copies/ml. Of the 347 participants in the same-day ART group, 277 (80%) were retained in care, 10 (3%) died, and 60 (17%) were LTFU. Among the 277 participants retained in the same-day ART group, 184 (66% of retained and 53% overall) had HIV-1 RNA <50 copies/ml. The unadjusted RR of being retained in care at 12 months and achieving HIV-1 RNA <50 copies/ml was 1.21 (95% CI: 1.04, 1.38; *p* = 0.015) for the same-day ART group compared to the standard group (Table 3); the adjusted RR for this comparison was 1.24 (95% CI: 1.06, 1.41; *p* = 0.008).

**Table 2:** Study outcomes by group.

Outcome	Standard ART Group (*n* = 356)	Same-Day ART Group (*n* = 347)	Unadjusted Risk Difference (95% CI)	*p*-value
**Primary Outcome**				
*Retained in care at 12 months with VL <50 copies/ml*	156 (43.8%)	184 (53.0%)	9.2% (1.8%, 16.6%)	0.015†
**Secondary Outcomes**				
*Retained in care at 12 months with VL <1*,*000 copies/ml*	184 (51.7%)	212 (61.1%)	9.4% (2.1%, 16.7%)	0.012‡
*Retained in care at 12 months*, *regardless of VL results*	256 (71.9%)	277 (79.8%)	7.9% (1.6%, 14.2%)	0.014††
*Died*	20 (5.6%)	10 (2.9%)		
*Lost to follow-up*	80 (22.5%)	60 (17.3%)		

† *p*-value comparing the proportion of all patients who were retained in care with viral load <50 copies/ml between the 2 arms.

‡ *p*-value comparing the proportion of all patients who were retained in care with viral load <1,000 copies/ml between the 2 arms.

†† *p*-value comparing the proportion of all patients who were retained in care between the 2 arms.

ART, antiretroviral therapy; VL, viral load.

**Table 3:** Unadjusted and adjusted risk ratios of study outcomes.

	Unadjusted			Adjusted for All Baseline Co-variates		
	RR	95% CI	*p*-value	RR	95% CI	*p*-value
	*Retained in care with viral load <50 copies/ml*					
**Standard ART Group**	1.0			1.0		
**Same-Day ART Group**	1.21	(1.04, 1.38)	0.015	1.24	(1.06, 1.41)	0.008
	*Retained in care with viral load <1*,*000 copies/ml*					
**Standard ART Group**	1.0			1.0		
**Same-Day ART Group**	1.18	(1.04, 1.31)	0.012	1.20	(1.05, 1.33)	0.008
	*Mortality during study period*					
**Standard ART Group**	1.0			1.0		
**Same-Day ART Group**	0.51	(0.24, 1.08)	0.073	0.43	(0.19, 0.94)	0.033

ART, antiretroviral therapy; RR, risk ratio.

In the standard ART group, 184 (72% of retained and 52% overall) participants who were retained in care had HIV-1 RNA <1,000 copies/ml. In the same-day ART group, 212 (77% of retained and 61% overall) participants who were retained in care had HIV-1 RNA <1,000 copies/ml. The unadjusted RR of being retained in care at 12 months and achieving HIV-1 RNA <1,000 copies/ml was 1.18 (95% CI: 1.04, 1.31; *p* = 0.012) for the same-day ART group compared to the standard ART group (Table 3); the adjusted RR for this comparison was 1.20 (95% CI: 1.05, 1.33; *p* = 0.008). In the sensitivity analysis that included only participants who met the original enrollment criteria (CD4 count ≤350 cells/mm^3^), the adjusted RR of being retained in care at 12 months and achieving HIV-1 RNA <50 copies/ml was 1.19 (95% CI: 0.99, 1.38; *p* = 0.060), and the adjusted RR of being retained in care at 12 months and achieving HIV-1 RNA < 1,000 copies/ml was 1.18 (95% CI: 1.01, 1.34; *p* = 0.035).

**Fig 3:**
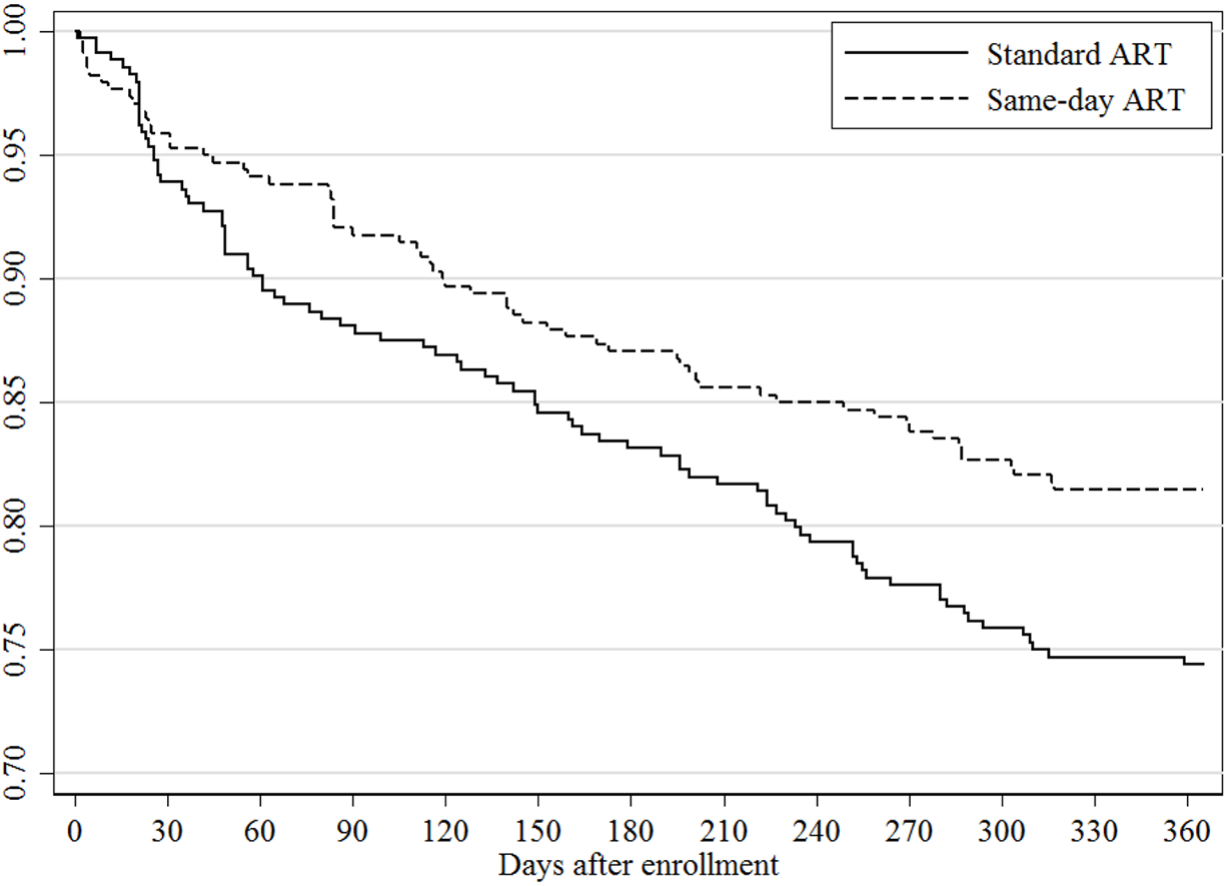
Retention in care by study group.

Vital status at the end of the study was known for 328 (92%) participants in the standard ART group and 329 (95%) in the same-day ART group. The unadjusted RR for mortality was 0.51 (95% CI: 0.24, 1.08; *p* = 0.073) for the same-day group compared to the standard group; the adjusted RR for this comparison was 0.43 (95% CI: 0.19, 0.94; *p* = 0.033). In the sensitivity analysis that included only participants with CD4 count ≤350 cells/mm^3^, the adjusted RR for mortality was 0.41 (95% CI: 0.18, 0.93; *p* = 0.033). Among the participants who died, the median baseline CD4 count was 100 cells/mm^3^ (IQR: 45, 192) in the standard and 207 cells/mm^3^ (IQR: 112, 291) in the same-day ART group (*p* = 0.078). Eight of 20 (40%) deaths in the standard ART group occurred in participants who were LTFU prior to ART, 8 (40%) deaths occurred in those LTFU after starting ART, and 4 (20%) occurred while in care; the causes of death for those in care were stroke, trauma, and cancer in 3, and the fourth had pain and died after seeing a traditional healer. Three of the 10 (30%) deaths in the same-day ART group occurred in participants who were LTFU after starting ART; among the 7 (70%) participants who died while in care, 1 of each died of stroke, pneumonia, malaria, renal failure, and sudden death, and 2 died of gastroenteritis. No deaths for those in care were attributed to immune reconstitution syndrome or an opportunistic infection that was missed at ART initiation. In Fig 3, the Kaplan-Meier curve plots the retention in care, regardless of viral load, for both groups. The log-rank test comparing the curves between the standard and same-day ART group indicates a significant difference (*p* = 0.028).

In the same-day ART group, 344 of 347 (99%) participants started ART on the day of HIV testing, and the remaining 3 patients started ART within the subsequent week. During the Day 3 follow-up visit, 13 patients (4%) in the same-day ART group had adjustments in their ART regimens (replacement of tenofovir with zidovudine or abacavir) because they had creatinine clearance <50 mL/minute on baseline testing. In the standard group, 281 (79%) participants initiated ART by Day 28, the end of the time window for the 3-week ART initiation visit. Thirty-six (10%) standard group participants initiated ART from Day 29 to Day 90, and 12 (3%) initiated ART after Day 90 due to late or missed visits. Twenty-seven (8%) standard group participants never started ART during the study period because they were LTFU or died prior to initiating treatment. Isoniazid prophylaxis was initiated for 337 (95%) participants in the standard group and 340 (98%) in the same-day group. Eight cases of TB were diagnosed during the first 3 months after ART initiation; 6 of these occurred in the standard group and 2 in the same-day ART group.

## Discussion

The results of this randomized controlled trial show that among HIV-infected adults with early WHO Stage disease and CD4 count ≤500 cells/mm^3^, same-day HIV testing and ART initiation, as compared to standard care, improves retention in care with virologic suppression and, in the multivariable analysis, decreases mortality. These results are important given recent WHO 2016 guidelines stating the lack of evidence in support of same-day ART initiation.

Our findings suggest that ART initiation as soon as possible after HIV testing may be beneficial for clinically stable patients. In resource-poor settings with fragile delivery systems, such as Haiti, the provision of immediate support by care providers at the time of HIV diagnosis can have both structural and individual impact. In addition to making treatment initiation logistically easier for patients, we believe that same-day counseling and ART initiation increase the sense of hope, optimism, and overall connectedness to the healthcare system for patients, which have been shown to be important for retention [17–20].

Our findings are consistent with the results of the RapIT study, a randomized trial that included participants in South Africa with WHO Stage 3 or 4 disease or CD4 count ≤350 cells/mm^3^ [11]. Participants in the standard group in that study generally started ART at the sixth visit, and 72% of participants in the rapid group started ART on the day of study enrollment. Rapid ART initiation resulted in a 17% improvement in retention and 13% improvement in viral suppression. A stepped-wedge cluster-randomized trial in Uganda found an increase in ART initiation within 2 weeks after eligibility by implementing a multicomponent intervention to streamline ART initiation that included training healthcare workers, providing point-of-care CD4 count testing platforms, eliminating mandatory multiple preinitiation sessions, and giving feedback to facilities on their ART initiation rates [21]. A weighted proportion of 80% in the intervention group had started ART within 2 weeks after eligibility compared with 38% in the control group. A cohort study of same-day ART initiation in pregnant women in South Africa also found high rates of treatment initiation, with 91% initiating ART on the day of referral to the service [22]. In the intervention group of the Sustainable East Africa Research on Community Health (SEARCH) HIV test-and-treat study, a cluster-randomized controlled trial conducted in Kenya and Uganda, HIV-infected patients who were identified through community testing were referred to HIV care upon diagnosis and then offered immediate ART initiation; retention was high (89%) among patients newly linking to care [23].

At ART initiation, it is critical that patients are ready to start lifelong therapy, that TB screening is conducted, and that renal function is evaluated to avoid the use of tenofovir in patients with renal insufficiency. In this study, ART readiness was remarkably high, with over 99% of patients screened for the study reporting they were ready to start lifelong ART. This is a particularly significant and timely finding for the provision of recommended universal ART because the majority of people living with HIV have early clinical disease, and there has been prior concern that healthier patients may be less willing to accept lifelong therapy [4]. Most patients with early clinical disease do not have TB symptoms (cough, fever, night sweats, or weight loss), so they do not require further work up to exclude TB, according to WHO guidelines [2]. With the exclusion of patients with a baseline chest x-ray that was suspicious for TB, we found that less than 1% of participants in the same-day ART group had TB that was missed at the time of ART initiation. We found that 4% of participants in the same-day ART group had creatinine clearance <50 mL/minute; ART regimens were adjusted on Day 3 for these patients.

Both groups in our study received high-level care, with multiple counseling and physician visits in the first month, followed by monthly physician visits. At the time the study was started, this was the standard of care in Haiti. However, this standard has shifted over the past few years towards decreased frequency of visits and nonphysician providers [2,24–27]. We believe that same-day ART can be provided with fewer follow-up visits if proper counseling is provided during the early period after ART initiation. However, clinic-level procedures play a major role in the effectiveness of accelerated ART initiation strategies, as illustrated in Malawi, where among nearly 22,000 pregnant women who started ART for mother-to-child prevention, LTFU rates ranged from 0% to 58% between facilities and were highest among women who initiated ART on the day of HIV testing at large clinics [28].

Though lower than anticipated, retention in both groups in our study was higher than reports of standard ART initiation from other resource-poor settings. Two studies from South Africa found that approximately one-third of patients remained in care from HIV testing through 12 months of ART, and systematic reviews of African studies have found high rates of pre-ART attrition [6,8,29,30]. In Haiti, data on pre-ART outcomes are limited, but 12-month retention after ART initiation is 73% nationwide [31]. We attribute the higher retention in our study in large part to faster ART initiation, even in the standard group, compared to many other HIV programs. We surmise that retention would have been lower in the standard group if there had been longer delays in ART initiation [5,11,30].

The rates of retention with viral suppression in our study are lower than those reported from clinical trial cohorts, including at GHESKIO. In the GHESKIO Clinical Trials Unit, with a median monthly average of 483 subjects participating in NIH-funded clinical trials, retention is 97%. We attribute the lower retention and viral suppression rates in our study to 2 major reasons. First, nearly all patients meeting WHO stage and CD4 criteria were enrolled in the study on the day of HIV testing, including those with substantial barriers to retention in care and adherence. In contrast, over one-third of patients are generally lost to care prior to ART initiation or enrollment in clinical trials [6,8,29,30]. Second, the care that was provided in this study was similar to that received by nonstudy patients at GHESKIO, with the aim of producing findings that could be reproduced in other resource-poor settings. In order to achieve the UNAIDS 90-90-90 targets, it will be important to evaluate reasons for attrition and implement new strategies to improve retention in care. One approach that has been successful in a cohort of nonresearch patients at GHESKIO has been expedited follow-up care, with fewer visits of shorter duration for clinically stable patients [32]. Streamlined care has also been associated with high rates of retention in the SEARCH study, which is described above [23].

Our study was conducted in a large urban clinic, which may limit the generalizability of our findings. In addition, though our study included patients with early clinical disease, the CD4 counts in our population were lower than would be expected with the provision of universal ART. It is possible that patients with higher CD4 counts may experience less benefit from same-day ART. It is also noteworthy that we conducted a chest x-ray prior to enrollment; if same-day ART is provided without a chest x-ray, it is possible that TB cases will be missed. Our study was not blinded. All participants in both groups received the same number of visits and the same retention plan, but we cannot exclude the possibility that awareness of study group impacted provider behavior.

In conclusion, in a population of asymptomatic or minimally symptomatic HIV-infected patients, same-day HIV testing and ART initiation decreased mortality and improved the rate of retention in care with virologic suppression compared with standard ART initiation. Furthermore, human and material resources provided to each group were similar, so same-day ART is not expected to increase treatment costs. The new WHO recommendations to provide ART to all HIV-infected patients should facilitate same-day test and treat.

## Supporting information

S1 Text Study protocol. (DOCX)

S2 Text CONSORT checklist. (DOC)

S3 Text HIV medication readiness scale. (PDF)

S1 Data Anonymized dataset. (XLSX)

## Abbreviations

ART: antiretroviral therapy
GHESKIO: Haitian Group for the Study of Kaposi’s Sarcoma and Opportunistic infections
IQR: interquartile range
LTFU: lost to follow-up
PPD: purified protein derivative
RR: risk ratio
SEARCH: Sustainable East Africa Research on Community Health
UNAIDS: The Joint United Nations Programme on HIV/AIDS
WHO: World Health Organization
