# Supplementary material for: Same-day HIV testing with initiation of antiretroviral therapy versus standard care for persons living with HIV: A randomized unblinded trial
Source: PLoS Med. 2017 Jul 25;14(7):e1002357. doi: 10.1371/journal.pmed.1002357 (PMC5526526; doi:10.1371/journal.pmed.1002357)
Supplement: S1 Text — (DOCX) [file pmed.1002357.s001.docx]

**R01AI104344-01A1**

Same-Day HIV Testing and Treatment Initiation to Improve Retention in Care

Sponsored by:

The National Institute of Allergy and Infectious Diseases

Principal Investigator: Serena Koenig, MD, MPH

Version 5.0

January 13, 2016

TABLE OF CONTENTS Page

STUDY SCHEMA……………………………………………………………………………….. 4

1.0 HYPOTHESIS AND SPECIFIC AIMS..……..………………………………………... 6

2.0 INTRODUCTION.…………………………………………………………………….… 7

2.1 Haitian Study Group for Kaposi’s Sarcoma and Opportunistic Infections.. 7

2.2 Historical Background…………………………………………………………. 8

2.3 Preliminary Data……………………………………………………………….. 10

2.4 Rationale………………………………………………………………………... 12

3.0 STUDY DESIGN………………………………………………………………………… 12

4.0 SELECTION AND ENROLLMENT OF SUBJECTS…………………………………. 13

4.1 Inclusion Criteria………………………………………………………………... 13

4.2 Exclusion Criteria……………………………………………………………….. 13

4.3 Source of Subjects and Recruitment Methods.…………....………….……. 13

4.4 Study Enrollment……………………………………………………………….. 14

4.5 Procedures for Obtaining Informed Consent……………………………….. 14

4.6 Treatment Assignment and Randomization………………………………… 15

5.0 STUDY INTERVENTIONS……………………………………………………………. 15

5.1 Study Visits and Parameters to be Measured……………………………… 15

5.2 Study Medications…………………………………………………………….. 18

5.3 Study Devices and Procedures/Surgical Interventions……………………. 18

6.0 CLINICAL AND LABORATORY EVALUATIONS…………………………………… 18

7.0 STATISTICAL CONSIDERATIONS………………………………………………….. 19

7.1 Study Endpoints………………………………………………………………. 19

7.2 Sample Size and Accrual…………………………………………………….. 20

7.3 Analyses……………………………………………………………………….. 21

8.0 DATA COLLECTION, MONITORING AND ADVERSE EVENT REPORTING … 22

8.1 Records to be Kept…………………………………………………………… 22

8.2 Data Management and Security…………………………………………….. 23

8.3 Monitoring and Quality Assurance…………………………………………... 24

8.4 Adverse Event Reporting…………………………………………………….. 24

8.5 Institutional Review Board Review and Informed Consent…………..…… 25

8.6 Subject Confidentiality………………………………………………………… 25

8.7 Data Safety Monitoring Board………………………………………………… 26

8.8 Study Discontinuation…………………………………………………………. 27

9.0 RISKS AND DISCOMFORTS………………………………………………………… 27

9.1 Complications of Surgical and Non-surgical Procedures…………………. 27

9.2 Drug Side Effects and Toxicities……………………………………………… 27

- 1. Device Complications/Malfunctions………………………………………….. 27
  2. Psychosocial (Non-Medical Risks)…………………………………………… 27

10.0 POTENTIAL BENEFITS………………………………………………………………... 28

10.1 Potential Benefits to Participating Individuals……………………………….. 28

- 1. Potential Benefits to Society…………………………………………………… 29

11.0 STUDY REMUNERATION……………………………………………………………… 29

12.0 PUBLICATION OF RESEARCH FINDINGS..……………………………………….. 29

13.0 REFERENCES………………………………………………………………………….. 30

APPENDIX 1 – INFORMED CONSENT……………………………………………………… 36

STUDY SCHEMA

TITLE: Same-Day HIV Testing and Treatment Initiation to Improve Retention in Care

DESIGN: Randomized, open label, clinical trial comparing standard and same-day ART initiation for patients who present for HIV testing with WHO stage 1 or 2 conditions, and a CD4 cell count ≤500 cells/mm^3^. In a sub-study, patients with WHO stage 1 or 2 conditions and CD4 count >500 cells/mm3 will be randomized to same-day ART vs. standard pre-ART care.

HYPOTHESIS: We hypothesize that the proportion of participants that are alive and in care with an undetectable viral load 12 months after HIV testing will be higher in the same-day ART group, that adherence will be the same or better, and that same-day ART will be more cost-effective than standard care. In a sub-study, we hypothesize that retention in care 6 months after HIV testing will be higher with same-day ART, compared with standard pre-ART care.

STUDY SITE: The study site is the GHESKIO Centers, Port-au-Prince, Haiti

POPULATION: The study population includes HIV-infected men and women ≥18 years of age who are ART-naïve, who present for HIV testing with WHO stage 1 or 2 conditions, and a CD4 cell count ≤500 cells/mm^3^. In a sub-study, patients with WHO stage 1 or 2 conditions and CD4 count >500 cells/mm^3^ will be included.

DURATION: Participants will be followed for 12 months after HIV testing. We estimate that enrollment (698 participants) will take approximately 12 months. In a sub-study, participants with CD4 count >500 cells/mm^3^ will be followed for 6 months. We estimate that enrollment for the sub-study (176 patients) will take 6 months. To account for transfers (patients who have left the country or moved to the countryside) – we have inflated our sample size, and plan to enroll a total of 938 patients between the main study and the sub-study.

SAMPLE SIZE: We calculated the sample size using the two-sided comparison of proportions. We estimate that 35% of participants in the standard group and 25% in the same-day ART group will have died, become lost to care, or have an HIV-1 RNA level ≥50 copies/ml at 12 months after HIV testing. There will be 80% power at a 0.05 significance level to detect this difference with 349 participants per arm, or 698 participants in the total cohort.  Since participants who transfer to another clinic will be excluded from the analysis, we will inflate our sample size to account for transfers. For the sub-study of patients with CD4 count >500 cells/mm^3^, we calculated the sample size using the two-sided comparison of proportions. We estimate that 20% of participants in the standard group and 5% in the same-day ART group will have died or become lost to care at 6 months after study enrollment. There will be 80% power at a 0.05 significance level to detect this difference with 88 participants per arm, or 176 participants in the total cohort. We will inflate the total number of participants we plan to enroll to 938 for the main study and sub-study, which considers a transfer rate of 7%.

RANDOMIZATION: Participants who meet study criteria and sign the informed consent will be randomized to standard or same-day ART in a 1:1 ratio using a computer-generated random-number list. Participants who are enrolled in the sub-study of patients with CD4 count >500 cells/mm^3^, will be randomized to standard pre-ART care or same-day ART in a 1:1 ratio using a random-number list as well.

INTERVENTION: *Overview of Standard Treatment:* Participants will have a total of 3 pre-ART visits with a physician for baseline testing (purified protein derivative [PPD], complete blood count [CBC], creatinine, alanine aminotransferase [ALT], aspartate aminotransferase [AST]) and other tests as clinically indicated. They will also have 3 scheduled visits with a social worker for ART literacy and adherence counseling. Participants will then start ART. They will also attend a scheduled physician visit and social worker visit 2 weeks post-ART initiation and scheduled physician visit 4 weeks after ART initiation. Pharmacists will provide adherence counseling at every ART visit.

*Overview of Same-Day ART*: Participants who are randomized to the same-day ART group will receive the same baseline tests as the standard group (PPD, CBC, creatinine, ALT, AST), and other tests as clinically indicated. They will also receive adherence support from the social worker, study physician and pharmacist, and they will start ART on the day of HIV testing. They will have a follow-up appointment on day 3, which will include a medical evaluation by the study physician and ART literacy/adherence counseling from a social worker. Weekly follow-up visits will be scheduled with the physician and social worker for the 3 subsequent weeks. Pharmacists will provide adherence counseling at every ART visit.

*Sub-Study – Standard Pre-ART Care:* Participants will receive HIV testing and blood draw for CD4 count. They will be enrolled on the same day they receive CD4 count results (≤7 days after the date of HIV testing). All participants will receive prophylactic treatment with trimethoprim-sulfamethoxazole and isoniazid, and a daily multivitamin, as is standard of care at GHESKIO. Participants will receive standard GHESKIO pre-ART care, which includes a monthly visit with a physician for 3 months, and then every other month visits. They will have a CD4 count annually, and start ART when they meet WHO criteria.

*Sub-Study – Same-Day Test and Treat*: Participants will receive testing and clinical care as described above for the same-day ART group. They will be enrolled in the study on the same day they receive CD4 cell count results (≤7 days after HIV testing). They will receive the same treatment protocol described above for patients in the same-day ART arm. Participants who are clinically stable, asymptomatic, and adherent at week 12 will qualify for expedited care at future visits, which includes dispensing of ART directly by nurses.

OUTCOMES Primary Outcome: Proportion of participants who are alive and in care with a plasma HIV-1 RNA level <50 copies/ml at 12 months after HIV testing (Aim 1). The primary outcome for the sub-study is the proportion of participants who are alive and in care at 6 months after study enrollment.

Secondary Outcomes: Secondary outcomes include only the main study, unless specified to include the sub-study.

1. Proportion of participants who are alive and in care with a plasma HIV-1 RNA level <200 copies/ml and <1000 copies/ml at 12 months after HIV testing.
2. Proportion of participants with >95% adherence during the first six months of ART, as measured by medication possession ratio (using pharmacy refill records) among those with at least six months of follow-up. In the sub-study of patients with CD4 count >500 cells/mm^3^, we will also measure the proportion of patients with >95% adherence to isoniazid and trimethoprim-sulfamethoxazole prophylaxis.
3. Proportion of participants with a plasma HIV-1 RNA level <50 copies/ml at six months after ART initiation, among those who are alive and in care;
4. Proportion of participants with a plasma HIV-1 RNA level <200 copies/ml and <1000 copies/ml at six months after ART initiation, among those who are alive and in care;
5. Proportion of participants who report zero missed doses on three-day self-report at six months after ART initiation, among those who are alive and in care;
6. Cost-effectiveness of standard and same-day ART from the health center perspective, as measured by the mean treatment cost per participant who is alive and in care with a plasma HIV-1 RNA level <50 copies/ml at 12 months after HIV testing. In a sub-study of patients with CD4 count >500 cells/mm^3^ we will measure the cost-effectiveness of standard and same-day ART at 6 months after study enrollment.
7. Cost-effectiveness of standard and same-day ART from the societal perspective, as measured by the mean treatment cost per participant who is alive and in care with a plasma HIV-1 RNA level <50 copies/ml at 12 months after HIV testing.
8. Proportion of participants in each group who initiate ART during the study period.
9. Proportion of participants in each group who are LTFU or died in the first month after study enrollment.
10. Median time to loss to follow-up or death in each group.
11. Proportion of participants who are in-care at one year, stratified by CD4 count at diagnosis.
12. Comparison of the standard and same-day ART groups to the non-research patients at GHESKIO regarding completion of CD4 count, timing of starting ART, loss to follow-up, and mortality.
13. Predictors of loss to follow-up and mortality in each of the two study groups, and among GHESKIO non-research ART patients.
14. Identify barriers and facilitators to clinic attendance and ART adherence through focus groups and in-depth interviews with patients in the standard care and same-day ART groups.

ANALYSES: *Aim 1 (Primary Aim):* We will compare the proportion of participants who are alive and in care with an HIV-1 RNA level <50 copies/ml in the standard and same-day ART groups at 12 months after HIV testing (binary outcome) using the two sample test of proportions. In a sub-study of patients with CD4 count >500 cells/mm^3^, we will compare the proportion of participants who are in-care at 6 months after HIV testing.

*Aim 2 (Secondary Aim):* We will use two methods to compare 6-month ART adherence between the standard and the same-day ART groups. First, we will measure adherence using the medication possession ratio. Six-month adherence will be measured by adding up the number of pills dispensed in the first 180 days, and dividing this by the number of pills that should have been dispensed in 180 days with perfect adherence. We will compare the proportion of participants in each group who received >95% of antiretroviral medications in the first 180 days of ART using the two-sample test of proportions. We will also use the two-sample test of proportions to compare the proportion of participants in each group who report perfect adherence (missing 0 doses in the prior 3 days) on the questionnaire of self-reported 3-day adherence described above. In the sub-study of patients with CD4 count >500 cells/mm^3^, this method will be used to compare adherence to isoniazid and trimethoprim-sulfamethoxazole.

*Aim 3 (Secondary Aim):* We will compare the cost and cost-effectiveness of standard and same-day ART, where cost is measured by the mean treatment cost and effectiveness is measured by being alive and in care with a plasma HIV-1 RNA level <50 copies/ml at 12 months after HIV testing.

1.0 HYPOTHESIS AND SPECIFIC AIMS

We propose a randomized trial to establish the effectiveness of same-day antiretroviral therapy (ART) initiation for patients who present for HIV testing with WHO stage 1 or 2 disease and a point-of-care (POC) CD4 cell count that is ≤500 cells/mm^3^ at the GHESKIO Center in Port-au-Prince, Haiti. All participants in the same-day ART group will receive rapid HIV antibody testing, POC CD4 cell testing, clinically relevant testing for OIs, WHO staging, counseling and social support, and ART initiation on the day of presentation. The standard group will receive the same services as the same-day ART group (including POC CD4 cell testing) except that instead of same-day ART, they will receive the standard GHESKIO protocol of three sequential visits for ART readiness counseling and testing for OIs prior to ART initiation. For the same-day ART group, these activities will take place on the day of ART initiation and during the early period post-ART initiation. We hypothesize that the proportion of participants that are alive and in care with an undetectable viral load 12 months after HIV testing will be higher in the same-day ART group, that adherence will be the same or better, and that same-day ART will be more cost-effective than standard care. For the proposed sub-study, we hypothesize that the proportion of participants who are alive and in care at 6 months after enrollment will be higher in the same-day ART group, compared with standard pre-ART care.

We propose three specific aims:

Aim 1: To compare the proportion of participants in the standard and same-day ART groups that are alive and in care with a plasma HIV-1 RNA level <50 copies/ml at 12 months after HIV testing. *Hypothesis: The proportion of participants alive, in care, and with a plasma HIV-1 RNA level <50 copies/ml will be 65% in the standard group and 75% in the same-day ART group.*

*For the sub-study of patients with CD4 count >500 cells/mm3, the primary outcome will be to compare the proportion of patients in the standard pre-ART and same-day ART groups who are alive and in-care at 6 months after HIV testing.*

Aim 2: To compare adherence for the first six months of ART between the standard and same-day ART groups using pharmacy refill records among those participants with at least six months of follow-up. *Hypothesis: Adherence will be non-inferior in same-day ART group compared to the standard group.*

Aims 3: To compare the cost and cost-effectiveness of standard and same-day ART, where cost is measured by mean treatment cost and effectiveness is measured by being alive and in care with a plasma HIV-1 RNA level <50 copies/ml at 12 months after HIV testing. *Hypothesis: Same-day ART will be more cost-effective than standard treatment.*

# 2.0 INTRODUCTION

2.1 Haitian Study Group for Kaposi’s Sarcoma and Opportunistic Infections

Created in 1982, the Haitian Study Group for Kaposi’s Sarcoma and Opportunistic Infections (GHESKIO) is the oldest non-governmental organization working in HIV/AIDS care and research in the world. GHESKIO is located in Port-au-Prince, Haiti, and provides treatment free of charge to all who present for care. GHESKIO has three missions: clinical and implementation research, patient care, and training. It is the largest HIV treatment center in the Caribbean, supervising about 50% of HIV treatment in Haiti. At GHESKIO, HIV voluntary counseling and testing is integrated with services to diagnose and treat sexually transmitted infections, tuberculosis and other communicable infections, and a program of comprehensive HIV/AIDS treatment. GHESKIO has been an international leader in conducting HIV/AIDS research ever since they completed the first report of AIDS in a developing country nearly 30 years ago. GHESKIO has conducted clinical research on the natural history of AIDS, treatment and prophylaxis for enteric infections, role of sexually transmitted infections in HIV transmission, treatment and prophylaxis of tuberculosis, long-term outcomes with HIV treatment in pediatric, adolescent, and adult groups, timing of initiation of ART, and cost-effectiveness of HIV treatment strategies. GHESKIO is also designated as an international site in the National Institutes of Health HIV Vaccine Trials Network (HVTN) and the AIDS Clinical Trials Group (ACTG), with multiple ongoing studies for these networks at this time.

2.2 Historical background

All published studies from resource-poor settings that have evaluated patient outcomes prior to initiation of ART have documented high rates of attrition at every step from HIV testing to ART initiation – see Table 1.[^1-12^](#_ENREF_1) Pre-ART attrition rates are much higher than on-ART attrition rates, and high mortality rates have been reported among qualifying patients who are lost to follow-up (LTFU) prior to ART initiation.[^6^](#_ENREF_6)^,^[^7^](#_ENREF_7)^,^[^9^](#_ENREF_9)^,^[^11^](#_ENREF_11)^,^[^13-17^](#_ENREF_13)

**Table 1: Pre-ART Attrition Rates among Patients who Qualify for Treatment**

| First Author and Year | Country | LTFU Prior to CD4 | CD4 <200 but LTFU pre-ART | Median time from screening to ART |
| --- | --- | --- | --- | --- |
| Mulissa, 2010 | Ethiopia | * | 25% | 16 days |
| Noel, 2012 | Haiti | 23% | 23% | 19 days** |
| McGrath, 2010 | Malawi | * | 14% | 22 days |
| Lawn, 2006 | South Africa | * | 19% | 29 days |
| Amuron, 2009 | Uganda | * | 26% | 33 days |
| Micek, 2009 | Mozambique | 23% | 69% | 71 days** |
| Ingle, 2010 | South Africa | 26% | 32% | 95 days |
| Bassett, 2009 | South Africa | * | 16% | 108 days*** |
| Bassett, 2010 | South Africa | 31% | 61% | 6.6 months** |
| Kranzer, 2010 | South Africa | 37% | 33% | * |
| Pepper, 2011 | South Africa | * | 34% | 58 days¥ |
| Larson, 2010 | South Africa | 46% | * | * |
| Losina, 2010 | South Africa | 45% | * | * |
| McGuire, 2010 | Malawi | 61% | * | * |
| Zachariah, 2011 | Malawi | * | 23% | 12 days |
| Zachariah, 2011 | Kenya | * | 15% | 27 days |

*Data not reported in study; **Median time from HIV testing to ART initiation; ***Mean time from CD4 cell count to first of three ART training visits; ¥ TB treatment until ART initiation

Treatment readiness is critical for long-term ART adherence. The 2010 World Health Organization (WHO) guidelines state that preparedness is a major factor contributing to good adherence, but they do not detail what constitutes treatment readiness.[^18^](#_ENREF_18) The standard of care in resource-poor settings is to clinically evaluate patients with newly diagnosed HIV and prepare them for ART over a period of several weeks. Many treatment programs require attendance in at least three ART literacy training sessions before ART is initiated. Requirements for multiple pre-ART literacy visits were developed at a time when there was concern that scaling up ART in resource-poor settings would be associated with poor adherence, but these fears have been dispelled, as recorded adherence in these settings is equal to or superior to that of industrialized countries.[^19^](#_ENREF_19)^,^[^20^](#_ENREF_20) This non-evidence-based strategy may discourage patients from accessing care, particularly because cumbersome pre-ART procedures and transportation fees are a barrier to ART initiation. Our proposed same-day ART approach will decrease barriers to ART initiation and may improve retention in care. We will shift the ART readiness activities that usually take place in the pre-ART visits to the day of ART and early period post-ART initiation. The study findings will also be relevant to the United States, as approximately 25% of patients with newly diagnosed HIV in the U.S. do not link to HIV care within the subsequent 6 to 12 months, and over 40% of known HIV-infected persons are not engaged in regular HIV care.[^21^](#_ENREF_21)

Our same-day ART approach is different from “Test and Treat” strategies, which involve early identification and ART initiation for all HIV-infected individuals (regardless of clinical or immunologic status) to reduce HIV transmission to others. “Test and Treat” strategies do not include same-day ART, and they are not focused on identifying patients who qualify immediately for ART by current guidelines. Our approach is also different from recommendations for “immediate” or “early” ART initiation for patients with TB and other WHO stage 3 and 4 conditions. Such recommendations refer to ART initiation within a few weeks after HIV diagnosis; there have been no studies to date evaluating ART initiation on the day of HIV testing.

Pima (Alere Inc., Waltham, MA) is a point-of-care CD4 cell test that has recently become available, with reported results that are accurate, precise, and unbiased when conducted in an accredited laboratory with venous whole blood specimens.[^22-24^](#_ENREF_22) A small sample of whole blood is collected into a disposable test cartridge, which is then inserted into the machine. All reagents are sealed into the test cartridge, and the sample is processed automatically, with results available within 20 minutes (see demonstration video at: <http://pimatest.com/en/pima-platform/what-is-pima.html>. The machine runs on external power or a rechargeable battery. (We note that GHESKIO has a generator and a back-up generator in order to maintain a reliable electrical source). The coupling of rapid HIV testing and point-of-care CD4 cell testing provides an opportunity to diagnose HIV and assess ART eligibility within a single visit. There are published reports describing lower attrition prior to CD4 cell testing when point-of-care tests are utilized, but attrition from CD4 cell completion to ART initiation remained high; this ongoing pre-ART attrition was attributed in part to barriers patients must overcome to attend multiple pre-ART visits.

Adherence in the early period after ART initiation must be emphasized, as it is a strong predictor of immunologic and virologic outcomes and long-term survival.[^19^](#_ENREF_19)^,^[^25-27^](#_ENREF_25) However, reported ART adherence rates in resource-poor settings are equal or superior to industrialized settings, and basic patient readiness counseling can be provided quickly, particularly with ongoing counseling in the period shortly after ART initiation. Furthermore, adherence measures such as the medication possession ratio, which measures the proportion of time an individual has ART in their possession (number of days ART dispensed out of the number of days in a time interval), may be calculated from information routinely available in pharmacy records and used to identify individuals with poor adherence who would benefit from targeted interventions.[^28-30^](#_ENREF_28)

With the availability of more effective, convenient, and better-tolerated ART regimens, the management of ART has changed. In the U.S., ART is now recommended for all HIV-infected patients.[^31^](#_ENREF_31) This recommendation is based on growing evidence that untreated HIV infection is associated with development of non-AIDS-defining diseases, including cardiovascular, renal, liver, and neurologic complications, and malignancies, and that effective treatment reduces transmission.[^31-44^](#_ENREF_31) The most recent WHO guidelines recommend ART for patients with CD4 count ≤500 cells/mm^3^ or WHO stage 3 or 4 disease.[^45^](#_ENREF_45) However, an African study of newly infected patients found that the majority reached the WHO ART initiation threshold within the subsequent year.[^46^](#_ENREF_46) One concern about changing the WHO guidelines to recommend universal ART is the need to prioritize patients with more advanced disease, particularly as many patients in resource-poor settings present for testing with severe immunosuppression. Haiti has achieved universal access to ART for patients with CD4 count ≤350 cells/mm^3^, and is widely scaling up ART for those with CD4 count ≤500 cells/mm^3^.[^47^](#_ENREF_47) In addition, the proportion of patients starting ART with CD4 count <200 cells/mm^3^ at GHESKIO dropped from 63% in 2005 to 27% in 2012, which is attributed to expanded access to HIV testing and earlier initiation of ART.[^47^](#_ENREF_47)

- 1. Preliminary Data

*CIPRA HT 001 Randomized Trial and Cost-Effectiveness Analysis*: This NIH-funded study, conducted at GHESKIO, compared survival with early ART (within 2 weeks) and standard ART (ART initiated after CD4 cell count ≤200 cells/mm^3^ or AIDS-defining illness occurred) among participants with a CD4 cell count >200 and <350 cells/mm^3^ and no AIDS-defining illness.[^48^](#_ENREF_48) Of the 408 participants in the early treatment group, 383 (94%) continued in follow-up to the end of the study (median duration of 21 months), 6 (1%) died, and 19 (5%) were LTFU. Of the 408 participants in the standard treatment group, 367 (90%) continued in follow-up to the end of the study, 23 (6%) died, and 18 (4%) were LTFU. The unadjusted hazard ratio for the risk of death with standard treatment as compared with early treatment was 4.0 (95% confidence interval [CI]: 1.6 to 9.8). We conducted a cost-effectiveness analysis (CEA) in parallel with this randomized trial.[^49^](#_ENREF_49) The cost of all medications, tests, labor, hospital services, and overhead were calculated at the patient level. Each of the resources used by each participant on each day was multiplied by the unit cost of that item, then summed to determine total costs for the arm. The costs for each arm were divided by the number of participants in the arm to determine the mean cost per participant for the duration of the trial. The mean survival time was estimated by the area under the Kaplan-Meier curve and the mean cost was estimated by the non-parametric method of Zhao and Tian in order to account for censoring. After excluding protocol-related tests, total treatment costs were $1,158 for early ART and $979 for standard ART. The cost-effectiveness ratio after a maximum of 3 years for early versus standard ART was $2,050 per year of life saved (YLS); the 95% confidence interval was $722/YLS to $5,537/YLS. We concluded that initiating ART in HIV-infected patients with a CD4 cell count between 200 and 350 cells/mm^3^ in Haiti, consistent with WHO advice, was cost-effective ($/YLS <3 times per capita GDP) after a maximum of 3 years after excluding protocol-related costs. The same method of calculating treatment costs will be used in the proposed research. *These data/experiences are important in establishing the feasibility of the specific aims, because they demonstrate that the study team has already conducted a large randomized trial and associated CEA.*

*Current Pre-ART Attrition at GHESKIO:*  From January 1 to June 30, 2009, 14,104 persons >13 years of age were tested for HIV at GHESKIO, and 1,427 patients (10%) tested positive.[^9^](#_ENREF_9) The median age was 34 years (interquartile range [IQR]: 27 to 42), and 62% were women. Fifty-six percent reported no school or primary school only, and 63% reported earning <$US125 per year. Of the 1,427 patients who tested HIV-positive, 1,083 (76%) received a CD4 cell count within the subsequent 12 months. Among the 1,083 patients who received a CD4 cell count, 110 patients (10%) did not return for the test result. Of the 973 patients who received CD4 cell count results, 297 (31%) had a CD4 cell count <200 cells/mm^3^, 247 (25%) had a CD4 cell count ≥200 and ≤350 cells/mm^3^, and 429 (44%) had a CD4 cell count >350 cells/mm^3^. Among the 544 patients with a baseline CD4 cell count ≤350 cells/mm^3^, 458 (84%) initiated ART at GHESKIO within the subsequent two years. We conducted bivariate and multivariate analyses of attrition prior to CD4 cell count, and attrition from CD4 cell count to ART initiation (for those with CD4 cell count <200 cells/mm^3^) using the following variables:  gender and active TB as binary variables; and age, education, annual income, residence zone, and baseline CD4 cell count as categorical variables. In multivariate analyses, attrition prior to CD4 cell testing was less common in older patients (age 35 to 44: OR 0.57; 95% CI: 0.38-0.84; and age ≥45: OR 0.51; 95% CI: 0.34-0.77), patients with higher income (>$US125: OR 0.66; 95% CI: 0.48-0.89) and patients with active TB at HIV testing (OR 0.08; 95% CI: 0.03-0.26) (Table 2). Among patients with CD4 cell count <200 cells/mm^3^, secondary or higher education (OR: 0.39; 95% CI: 0.21-0.74) and CD4 cell count 150 to 199 cells/mm^3^ (OR: 0.41; 95% CI: 0.18-0.89) were associated with lower odds of attrition prior to ART initiation (see Table 3). In contrast, attrition prior to ART initiation (OR: 2.46; 95% CI: 1.29-4.71) was more common among patients with active TB at the time of HIV testing. We attribute higher rates of CD4 cell testing but lower ART initiation among TB patients to faster services but higher pre-ART mortality[^9^](#_ENREF_9). In another study of 356 ART patients at GHESKIO, we found that 30% (n=107) waited more than six months to seek care after finding out their HIV diagnosis, and of these, 66% (n=71) delayed seeking care due to the logistics and costs of getting to clinic.

**Table 2. Risk Factors for Attrition Prior to CD4 Cell Count**

| Variable | Reference Group | Unadjusted OR (95% CI) | p-value | Adjusted OR (95% CI) | p-value |
| --- | --- | --- | --- | --- | --- |
| Age 25-34 years | ≤ 24 years | 0.84 (0.60-1.18) | 0.3118 | 1.01 (0.71-1.44) | 0.9627 |
| Age 35-44 years | ≤ 24 years | 0.47 (0.33-0.68) | <0.0001 | 0.57 (0.38-0.84) | **0.0046** |
| Age ≥ 45 years | ≤ 24 years | 0.44 (0.30-0.67) | <0.0001 | 0.51 (0.34-0.77) | **0.0015** |
| Income $US 1-125 | No income | 1.22 (0.89-1.68) | 0.2230 | 1.26 (0.90-1.76) | 0.1783 |
| Income >$US125 | No income | 0.61 (0.47-0.81) | 0.0007 | 0.66 (0.48-0.89) | **0.0064** |
| Active TB | Absence of TB | 0.08 (0.02-0.25) | <0.0001 | 0.08 (0.03-0.26) | **<0.0001** |

**Table 3. Risk Factors for Pre-ART Attrition with CD4 cell count <200 cells/mm^3^**

| Variable | Reference Group | Unadjusted OR (95% CI) | p-value | Adjusted OR (95% CI) | p-value |
| --- | --- | --- | --- | --- | --- |
| Some primary school | No school | 0.86 (0.45-1.66) | 0.6581 | 0.78 (0.40-1.53) | 0.4629 |
| Some secondary school | No school | 0.43 (0.23-0.80) | 0.0086 | 0.39 (0.21-0.74) | **0.0037** |
| Active TB | Absence of TB | 2.37 (1.27-4.44) | 0.0070 | 2.46 (1.29-4.71) | **0.0066** |
| CD4: 50-99 cells/mm^3^ | <50 | 0.93 (0.47-1.83) | 0.8264 | 0.88 (0.44-1.76) | 0.7172 |
| CD4: 100-149 cells/mm^3^ | <50 | 1.19 (0.60-2.38) | 0.6184 | 1.00 (0.49-2.05) | 0.9917 |
| CD4: 150-199 cells/mm^3^ | <50 | 0.49 (0.23-1.06) | 0.0693 | 0.41 (0.18-0.89) | **0.0252** |

*Presenting Symptoms in Study Population:* We evaluated symptoms at HIV testing among 500 randomly selected patients out of the 1,063 adults (age ≥18 years) newly diagnosed with HIV who received a CD4 cell count in the cohort described above.[^9^](#_ENREF_9) Among the 437 charts which were complete, including a physician intake evaluation, 144 (33%) of patients had WHO stage 1 or 2 disease and a CD4 cell count ≤350 cells/mm^3^. Of these, 62 patients (43%) were asymptomatic, and 82 patients (57%) presented with the following 112 illnesses: herpes zoster in 34 (30%), upper respiratory tract infection (URI) in 29 (26%), papular pruritic eruptions in 15 (13%), unexplained fever less than one month duration in 15 (13%), moderate unexplained weight loss in 11 (10%), gastrointestinal (GI) symptoms in five (4%), and lymphadenopathy in three (3%).

*Same-day ART Initiation at GHESKIO:* GHESKIO provides same-day ART initiation for patients who present with very severe clinical disease, due to the high risk of mortality without immediate treatment in this patient population.[^50^](#_ENREF_50) We conducted a pilot feasibility study of immediate ART initiation for 20 consecutively-enrolled patients with WHO stage 3 or 4 disease in March, 2010 (unpublished data). Twelve patients (60%) were female and the median age was 38 years. All patients were started on ART appropriately, with comprehensive counseling and medical assessments for OIs prior to ART initiation and in the early period after ART initiation. One patient moved to a distant city and transferred care. Of the other 19 patients, 17 (89%) were alive and in care 12 months after ART initiation, one patient was LTFU, and one patient died. We also note that in routine clinical care at GHESKIO, we start patients who present with severe HIV-associated TB on same-day ART. In our experience, we have found that immediate ART is feasible and well-tolerated in these patients. In the same pilot study, we included 15 patients with WHO stage 1 or 2 disease and CD4 cell count ≤350 cells/mm^3^. ART was started on the second visit (generally within 2 days after HIV testing) after results of CD4 cell testing were available. Ten patients (67%) were female; the median age was 39 years and median CD4 cell count was 235 cells/mm^3^. Two (13%) were asymptomatic, six (40%) had herpes zoster, three (20%) had GI symptoms, three (20%) had URIs, and one had moderate weight loss. At one year, 14 patients (93%) were alive and in-care, and one was LTFU.

## 2.4 Rationale

Further research to identify the most effective strategies to rapidly identify patients that qualify for ART, minimize barriers to ART initiation, and retain patients in care are critical to the success of HIV treatment programs worldwide. We hypothesize that the proportion of participants that are alive and in care with an undetectable viral load 12 months after HIV testing will be higher in the same-day ART group, that adherence will be the same or better, and that same-day ART will be more cost-effective than standard care. If the proposed research demonstrates that it is safe and effective to provide same-day ART, then other programs around the world are likely to consider the possibility that they could minimize their pre-ART requirements for ART literacy and adherence counseling visits and medical evaluations for OIs.

For the sub-study of patients with CD4 count >500 cells/mm^3^, the rationale is that further research to identify the most effective strategies to retain patients in care is critical to the success of HIV treatment programs worldwide. We hypothesize that the proportion of participants who are alive and in care at 6 months after study enrollment will be higher in the same-day ART group, compared with the standard pre-ART group.

# 3.0 STUDY DESIGN

*Study Design*: Randomized, open label, clinical trial comparing standard and same-day ART initiation for patients who present for HIV testing with WHO stage 1 or 2 conditions, and a CD4 cell count ≤500 cells/mm^3^ with point-of-care CD4 cell testing. A sub-study will include patients with WHO stage 1 or 2 conditions and a CD4 count >500 cells/mm^3^; patients will be randomized to standard pre-ART care or same-day ART.

*Study Site and Population*: The study site is the outpatient HIV clinic of the GHESKIO Center in Port-au-Prince, Haiti. All patients testing for HIV will receive same-day test results. They will be offered point-of-care CD4 cell testing. HIV-infected men and women ≥18 years of age who have CD4 cell count ≤500 cells/mm^3^ will be provided with an immediate physician evaluation, since they qualify for ART. If they meet study criteria, the study staff will provide them with information on the study. A sub-study will include patients with CD4 count >500 cells/mm^3^. They will be enrolled on the day they receive their CD4 count result, which will take place within 7 days of HIV testing.

4.0 SELECTION AND ENROLLMENT OF SUBJECTS

## 4.1 Inclusion Criteria

- Age ≥18 years;
- CD4 cell count ≤500 cells/mm^3^;
- Ability and willingness of participant to give written informed consent;
- WHO stage 1 or 2 disease as defined by the following conditions:
  - Asymptomatic
  - Persistent generalized lymphadenopathy
  - Moderate unexplained weight loss (under 10% of presumed or measured body weight)
  - Recurrent upper respiratory tract infections (sinusitis, tonsillitis, otitis media, pharyngitis)
  - Herpes zoster
  - Angular cheilitis
  - Recurrent oral ulcerations
  - Papular pruritic eruptions
  - Seborrheic dermatitis
  - Fungal nail infections

4.2 Exclusion Criteria

- Any use of ART in the past;
- Pregnancy or breastfeeding at the screening visit;
- Score of <3 for any of the 7 questions on the ART readiness survey, or failure to appropriately answer the 2 HIV knowledge questions;
- Patient plans to transfer care to another clinic during the study period;
- WHO stage 3 or 4 disease

The proposed sub-study will include identical inclusion and exclusion criteria, except that patients with WHO stage 1 or 2 disease and CD4 count >500 cells/mm^3^ will be enrolled in the study.

We will exclude patients that have had ART in the past, because including these patients may bias the study results. We will exclude patients that are pregnant or breastfeeding because they have a unique and urgent need for ART that should be addressed in a separate study. We will exclude patients with denial of HIV or who are not ready to start ART, because we do not believe that same-day ART would be safe for them due to the high likelihood of poor adherence.

4.3 Source of Subjects and Recruitment Methods

Subjects will be recruited from the GHESKIO HIV voluntary counseling and testing center. All patients testing for HIV will receive same-day test results. They will be offered point-of-care CD4 cell testing. Those who have CD4 cell count ≤500 cells/mm^3^ will be provided with an immediate physician evaluation, since they qualify for ART. If they meet study criteria, the study staff will provide them with information on the study. All adult patients (at least 18 years of age) who meet study criteria will be offered enrollment in the study. All patients at the clinic are Haitian; there will be no discrimination based on race, gender, educational status, or income. All subjects will speak Haitian Creole. All informed consent conversations, informed consent documents, and all study questionnaires will be conducted in Haitian Creole, as in all other NIH-funded studies at GHESKIO.

We do not plan to recruit any patients outside of the GHESKIO clinic. All patients that test positive for HIV and meet study criteria will be offered enrollment. Historically, 98% of patients accept study enrollment at GHESKIO. We do not anticipate that any further methods of recruitment will be necessary. GHESKIO tests about 28,000 adult patients per year for HIV; about 10% of patients (n=2800) test positive. Of these, about 70% have a CD4 cell count ≤500 cells/mm^3^. Our sample size is 698 participants excluding transfers; we anticipate enrollment will take about 12 months.

For the sub-study of patients with CD4 count >500 cells/mm^3^, the same recruitment methods will be used. The sample size for the sub-study is 176 patients; we anticipate enrollment will take about 6 months.

- 1. Study Enrollment

Patients who test HIV-positive will be offered a same-day CD4 cell count, and those with a CD4 cell count ≤500 cells/mm^3^ will receive a same-day evaluation by the study physician. The physician will conduct a complete physical examination and chest radiograph, and determine the patient’s WHO stage. Potentially eligible patients will be evaluated by the psychologist to ensure that they understand their HIV diagnosis. He/she will query the patient on two HIV knowledge questions (“I believe that HIV and AIDS exist” (Yes/No) and “I believe that the results of my HIV test show that I am infected” (Yes/No). The psychologist will educate the patient regarding ART, according to the established protocol. He/she will then administer an ART readiness scale that has been adapted from Balfour et al, translated into Creole, and validated for use in Haiti; the readiness scale will be administered orally in Creole. There are seven questions, such as: “If you were to start taking pills today to treat your HIV, how ready would you be to take the pills each day as prescribed, as close to the same time as possible.” Responses range from 0 (not ready) to 4 (completely ready). Patients that provide affirmative answers for both HIV knowledge questions and respond with “3” or “4” to each of the seven ART readiness questions will be told of the opportunity to enroll in a study, and referred to the study physician.

- 1. Procedures for Obtaining Informed Consent

The study staff will complete the informed consent process after determining that the patient meets study criteria. GHESKIO has a history of conducting clinical research for nearly 30 years, and has conducted several randomized trials. The GHESKIO staff has a long history of obtaining informed consent in similar study populations. An informed consent letter will be read aloud in Haitian Creole to all study subjects by the study staff, in a private examination room, where they cannot be overheard. The informed consent includes a clear description of the purpose of the study that is understandable in local terms and appropriate for patients that are not able to read or write; information on what is required for study participation; a detailed description of the risks and benefits involved with enrolling in the study; assurances of confidentiality and the voluntary nature of participation; and contact information of the PI, the GHESKIO Director, and the GHESKIO IRB in case the study participant has questions. The patient will be asked if he or she has any concerns, which will be answered by the study staff at the time of enrollment. If the person agrees to participate, he or she will then be asked to sign the informed consent. For those patients that cannot sign their name, a fingerprint will be accepted. The informed consent letter and the study protocol will be approved by all participating institutions prior to the start of the study.

For the proposed sub-study of patients with CD4 count >500 cells/mm^3^, a separate informed consent form will be used. Patients who decline study participation will be asked if they are willing to provide the reason they declined participation, and if they give permission for the study staff to review their medical information and include it in study analyses.

- 1. Treatment Assignment and Randomization

Participants who meet study criteria and sign the informed consent will be randomized to standard or same-day ART in a 1:1 ratio using a computer-generated random-number list, as has been done in prior GHESKIO studies. Participation in the study won’t impact the type of ART administered, or the dose or frequency. All participants will receive the same ART as standard GHESKIO patients – and all services are free for everyone that receives care at GHESKIO. The timing of initiation of ART for the standard group will be the current GHESKIO standard of care, which involves three visits with the physician and social worker prior to starting ART. The same-day ART group will initiate ART on the day of HIV testing. We have selected this one-day window because we are confidant that we can medically assess participants and provide adherence counseling in one day, and any additional delays create a barrier for patients to initiate ART. For the proposed sub-study, patients who are randomized to standard pre-ART care will receive the same care that is provided to patients in pre-ART care at GHESKIO. Patients who are randomized to the same-day ART group will receive care that is similar to that provided to the patients in the ongoing same-day ART study.

5.0 STUDY INTERVENTIONS

5.1 Study Visits and Parameters to be Measured

Overview of Standard Treatment: Participants will have a total of three pre-ART visits with a physician (as outlined in Table 4) for baseline testing (purified protein derivative [PPD], complete blood count [CBC], creatinine, alanine aminotransferase [ALT], aspartate aminotransferase [AST]) and other tests as clinically indicated. They will also have three scheduled visits with a social worker for ART literacy and adherence counseling. Participants will then start ART. They will attend additional visits with physicians and other staff as described in Table 4. These will include a scheduled physician visit and social worker visit two weeks post-ART initiation and scheduled physician visit four weeks after ART initiation. Pharmacists will provide adherence counseling at every ART visit.

**Table 4: Comparison of Care in the Standard and Same-Day ART Groups**

|  | **Standard Group** | **Same-Day ART Group** |
| --- | --- | --- |
| **Day 1** | | |
| VCT Social Worker and Lab Technician | HIV pre- and post-test counseling and CD4 cell count testing. | |
| Study Physician and Psychologist | Complete examination, chest radiograph, WHO staging, psychological assessment, pregnancy testing, and informed consent. | |
| Lab tests | Other tests as clinically indicated | Standard baseline tests (PPD; CBC, creatinine, ALT, AST); other tests as clinically indicated. |
| Study Social Worker | No scheduled visit | 1^st^ ART literacy/adherence visit |
| Study Pharmacist | No scheduled visit | Adherence counseling |
| ART Initiation | No | Yes |
| **From Day 2 until ART Initiation** | | |
| Physician | Two scheduled physician visits to provide test results, assess for OIs, and provide adherence counseling. | n/a |
| Additional Pre-ART Testing | Standard baseline tests (PPD; CBC, creatinine, ALT, AST), and other tests as clinically indicted. |  |
| Study Social Worker | 1^st^, 2^nd^, 3^rd^ ART literacy/adherence visits (3rd visit on day ART started) |  |
| ART Initiation | ART to be started after 3 pre-ART visits. |  |
| Study Pharmacist | Counseling on day ART started. |  |
| **Three Days Post-ART** | | |
| Physician | No scheduled visit | Medical assessment and adherence counseling |
| Social worker | No scheduled visit | 2^nd^ ART literacy/adherence visit |
| Pharmacist | No scheduled visit | Adherence counseling |
| **10 to 14 Days Post-ART** | | |
| Physician | Scheduled visit for medical assessment and adherence counseling | |
| Social worker | 4^th^ ART literacy/adherence visit | 3^rd^ ART literacy/adherence visit |
| Pharmacist | Adherence counseling | |
| **17 Days Post-ART** | | |
| Physician | No scheduled visit | Medical assessment and adherence counseling |
| Social worker | No scheduled visit | 4^th^ ART literacy/adherence visit |
| Pharmacist | No scheduled visit | Adherence counseling |
| **24 to 28 Days Post-ART** | | |
| Physician | Scheduled visit for medical assessment and adherence counseling | |
| Social worker | No scheduled visit | |
| Pharmacist | Adherence counseling | |
| **Adherence Support** | | |
| Social worker | Quarterly adherence reinforcement visits | |
| Missed visits | Phone call and/or home visits from CHWs | |
| Transportation Fees | Participants will be reimbursed for transportation fees at every visit. | |

Overview of Same-Day ART: As described in Table 4, participants who are randomized to the same-day ART group will receive the same baseline tests as the standard group (PPD, CBC, creatinine, ALT, AST), and other tests as clinically indicated. They will also receive adherence support from the social worker, study physician and pharmacist, and they will start ART on the day of HIV testing. They will have a follow-up appointment on day 3, which will include a medical evaluation by the study physician and ART literacy/adherence counseling from a social worker. Weekly follow-up visits will be scheduled with the physician and social worker for the three subsequent weeks. Pharmacists will provide adherence counseling at every ART visit.

Differences in Care between the Standard and Same-Day ART Groups: The only difference between the standard and same-day ART groups will be the timing of the services provided in the first month of follow-up. The standard group will receive the standard GHESKIO protocol of three sequential visits for ART readiness counseling and testing for OIs prior to ART initiation. For the same-day ART group, these activities will take place on the day of ART initiation and in the early period post-ART initiation. The number of physician and social worker visits and the content of all social worker counseling sessions will be the same between groups.

Additional Care for Both Groups: Prophylactic treatment with trimethoprim-sulfamethoxazole and isoniazid will be administered to all participants. After the first month of ART, participants in both groups will be seen monthly by a physician and receive the package of services provided to all HIV-infected patients at GHESKIO, which includes quarterly adherence reinforcement visits with a social worker. *Participants in either study group who are determined to be non-compliant will receive additional counseling sessions, as is standard of care at GHESKIO.* All clinical information will be entered into the GHESKIO electronic medical record (EMR), and the pharmacists will record the number of pills dispensed in the GHESKIO EMR each time they dispense ART. Plasma HIV-1 RNA levels (viral load) will also be measured (NucliSens EasyQ, BioMerieux, France) at six months post-ART initiation and at 12 months post-HIV testing for all participants who remain in care at those time points. Patients in either group with detectable viremia will receive antiretroviral drug resistance testing, changes to their ART regimen as clinically indicated, and additional counseling on ART adherence, as is standard of care at GHESKIO.

Sub-Study:

Overview of Supportive Care for Both Groups in the Sub-Study: Participants will be enrolled in the study on the same day they receive CD4 count results, which will be within 7 days of the date of HIV testing. All patients will receive additional tests as clinically indicated. All participants will receive prophylactic treatment with trimethoprim-sulfamethoxazole and isoniazid, and a daily multivitamin, as is standard of care at GHESKIO.

Standard Pre-ART Care: Participants will receive standard GHESKIO pre-ART care, which includes a monthly visit with a physician for 3 months, and then every other month physician visits. They will have a CD4 count annually, and start ART when they meet WHO criteria.

Same-Day ART: Participants will receive counseling and start ART on the day of study enrollment. They will have follow-up visits with the physician on Days 3, 10, 17, and 24, and with the social worker on Days 3, 10, and 17. They will have also have physician visits at weeks 7 and 12. Participants who are clinically stable, asymptomatic, and adherent at week 12 will then qualify for expedited care at future visits, which includes dispensing of ART directly by nurses in the clinic every four weeks, to reduce waiting time for patients. Participants who are symptomatic or non-adherent will be referred to a physician for evaluation.

ART Literacy and Adherence Counseling: Adherence counseling at GHESKIO has been developed over a decade of providing ART, and is based on a team approach involving the physician, pharmacist and social worker. This collaborative strategy was adapted from evidence-based approaches including cognitive behavioral therapy and motivational interviewing. Physicians devote five to ten minutes to providing adherence counseling in the first five visits, and provide reinforcement adherence counseling at all subsequent visits. The pharmacist provides about ten minutes of adherence counseling each time they dispense ART. The social worker provides more extensive adherence counseling in four ART literacy and adherence sessions that last about 30 to 45 minutes each. These counseling sessions follow a standardized protocol. The standard group will receive the first two sessions prior to starting ART, the third on the day of ART, and the fourth after two weeks of ART. The same-day group will receive these sessions on the day of ART initiation and in their first three ART visits. For this study, these sessions will be audiotaped for quality control purposes, and will include discussions of the following topics:

- Importance of building good communication and collaboration between the study participant and all members of the treatment team;
- Education about HIV/AIDS, modes of transmission and prevention;
- Definition of and education about ART adherence, consequences of non-adherence, barriers to adherence and strategies for overcoming them;
- Education about HIV medications, the specific ART regimen prescribed to the participant, including the dose, the timing of doses, food restrictions, if any, expected side effects and possible remedies;
- Pill storage and sorting;
- Development of plan for daily medication schedule, reminder strategies and plans to overcome anticipated obstacles;
- Discussion of family, community, social support, HIV disclosure, and privacy;
- Development of plans to handle different potential barriers to adherence and handling of slips and missed doses;
- Getting to appointments and plans for handling barriers to keeping appointments.

As a result of the adherence counseling, participants will be able to understand their own regimen, understand the importance of adherence to their health, and problem-solve with members of the treatment team about barriers and facilitators to adherence.

5.2 Study Medications

All ART provided will be standard as recommended by the guidelines of the WHO and the Haitian National AIDS Program. First-line therapy consists of tenofovir, lamivudine, and either efavirenz or nevirapine. Participation in this study will have no impact on the selection of the ART regimen.

5.3 Study Devices and Procedures/Surgical Interventions: None

6.0 CLINICAL AND LABORATORY EVALUATIONS

See Table 5 for a summary of clinical and laboratory monitoring tests. The medical history will include all past and present medical conditions, as well as HIV-related opportunistic infections. Any medication allergies will also be documented. The initial physical examination will include signs and symptoms; vital signs (temperature, pulse, respiration rate, blood pressure, height, and weight); examination of the skin, head, mouth, and neck; auscultation of the chest; cardiac exam; abdominal exam; and examination of the lower extremities for edema. At subsequent visits, a targeted physical examination will be conducted, which will include vital signs (temperature, pulse, respiration rate, and blood pressure) and will driven by any previously identified or new signs or symptoms including diagnoses that the subject has experienced since the last visit. For the proposed sub-study, the clinical and laboratory monitoring will be as listed in Table 5, except that follow-up will be 6 months in duration, and the standard pre-ART group will not receive viral load testing.

**Table 5: Schedule of Clinical and Laboratory Monitoring**

| Evaluation | Screening | Baseline Testing after Study Entry | Months 1 and 2 | Month 3 | Months 4 and 5 | Month 6 | Months 7 and 8 | Month 9 | Months 10 and 11 | Month 12 |
| --- | --- | --- | --- | --- | --- | --- | --- | --- | --- | --- |
| HIV Test | X |  |  |  |  |  |  |  |  |  |
| CD4 cell count | X |  |  |  |  |  |  |  |  |  |
| Medical History | X |  | X | X | X | X | X | X | X | X |
| Clinical Assessment | X |  | X | X | X | X | X | X | X | X |
| CXR | X |  |  |  |  |  |  |  |  |  |
| Pregnancy Test | X |  |  |  |  |  |  |  |  |  |
| ART Readiness Survey | X |  |  |  |  |  |  |  |  |  |
| Complete Blood Count |  | X |  |  |  |  |  |  |  |  |
| Liver Function Tests |  | X |  |  |  |  |  |  |  |  |
| Creatinine |  | X |  |  |  |  |  |  |  |  |
| PPD |  | X |  |  |  |  |  |  |  |  |
| Coping Survey |  | X |  | X |  | X |  | X |  | X |
| Adherence Questionnaire |  |  |  |  |  | X |  |  |  |  |
| HIV-1 RNA |  |  |  |  |  | X |  |  |  | X |

# 7.0 STATISTICAL CONSIDERATIONS

## 7.1 Study Endpoints

Primary Outcome: Proportion of participants who are alive and in care with a plasma HIV-1 RNA level <50 copies/ml at 12 months after HIV testing (Aim 1). For the sub-study, the primary outcome will be the proportion of participants who are alive and in care at 6 months after study enrollment.

Secondary Outcomes: Unless directly specified below, these aims will be conducted only for the main study.

1. Proportion of participants who are alive and in care with a plasma HIV-1 RNA level <200 copies/ml and <1000 copies/ml at 12 months after HIV testing.
2. Proportion of participants with >95% adherence during the first 6 months of ART, as measured by medication possession ratio (using pharmacy refill records) among those with at least six months of follow-up. For the sub-study of patients with CD4> 500 cells/mm^3^, adherence to isoniazid and trimethoprim-sulfamethoxazole will be measured.
3. Proportion of participants with a plasma HIV-1 RNA level <50 copies/ml at six months after ART initiation, among those who are alive and in care;
4. Proportion of participants with a plasma HIV-1 RNA level <200 copies/ml and <1000 copies/ml at six months after ART initiation, among those who are alive and in care;
5. Proportion of participants who report zero missed doses on three-day self-report at six months after ART initiation, among those who are alive and in care;
6. Cost-effectiveness of standard and same-day ART from the health center perspective, as measured by the mean treatment cost per participant who is alive and in care with a plasma HIV-1 RNA level <50 copies/ml at 12 months after HIV testing. For the sub-study of patients with CD4 >500 cells/mm^3^, the cost-effectiveness of standard pre-ART care and same-day ART for 6 months after HIV study enrollment
7. Cost-effectiveness of standard and same-day ART from the societal perspective, as measured by the mean treatment cost per participant who is alive and in care with a plasma HIV-1 RNA level <50 copies/ml at 12 months after HIV testing.
8. Proportion of participants in each group who initiate ART during the study period.
9. Proportion of participants in each group who are LTFU or died in the first month after study enrollment.
10. Median time to loss to follow-up or death in each group.
11. Proportion of participants who are in-care at one year, stratified by CD4 count at diagnosis.
12. Comparison of the standard and same-day ART groups to the non-research patients at GHESKIO regarding completion of CD4 count, timing of starting ART, loss to follow-up, and mortality.
13. Predictors of loss to follow-up and mortality in each of the two study groups, and among GHESKIO non-research ART patients.
14. Identify barriers and facilitators to clinic attendance and ART adherence through focus groups and in-depth interviews with patients in the standard care and same-day ART groups.

## 7.2 Sample Size and Accrual

We calculated the sample size for Aim 1 using the two-sided comparison of proportions. We anticipate a decrease in negative outcomes with same-day ART, but since this is a new approach to starting ART, we will look for both increases and decreases in negative outcomes. We estimate that 35% of participants in the standard group and 25% in the same-day ART group will have died, become lost to care, or have an HIV-1 RNA level ≥50 copies/ml at 12 months after HIV testing (see Table 6). There will be 80% power at a 0.05 significance level to detect this difference with 349 participants per arm, or 698 participants in the total cohort.

**Table 6: Predicted Patient Outcomes by Group**

|  | Historical GHESKIO | Standard Group | Same-Day Group |
| --- | --- | --- | --- |
| **Pre-ART initiation** |  |  |  |
| *Attrition from CD4 blood draw to CD4 completion* (A)* | 10% | 0% | 0% |
| *Attrition from CD4 completion to ART initiation* (B)* | 15% | 15% | 2% |
| Proportion of Patients Initiating ART *(C=100%-(A+B))* | 75% | 85% | 98% |
| **Post-ART initiation (for those who start ART)** |  |  |  |
| *Attrition from ART initiation through 12 months of follow-up (D)* [*^51^*](#_ENREF_51) | 15% | 15% | 15% |
| *Proportion of patients with HIV-1 RNA <50 copies/ml after 12 months follow-up (E)* [*^52^*](#_ENREF_52) | 90% | 90% | 90% |
| Alive and in care from ART initiation through 12 months of follow-up with HIV-1 RNA level <50 copies/ml *(F=(1-D)*E)* | 77% | 77% | 77% |
| **For all patients (Pre- and Post-ART initiation)** |  |  |  |
| Alive and in care from HIV testing through 12 months post-HIV test with HIV-1 RNA level <50 copies/ml *(G=C*F)* | 58% | 65% | 75% |
| Died, LTFU, or HIV-1 RNA level ≥50 copies/ml at 12 months after HIV test *(H=1-G)* | 42% | 35% | 25% |

*CD4 completion occurs when patient receives result of CD4; pre-ART attrition is 15% at GHESKIO if CD4 cell count ≤350 cells and WHO stage 1 or 2

For Aim 2 (adherence), we used a non-inferiority formula to calculate the sample size, as we hypothesize that adherence will be the same or better with same-day ART.  If we can show that we are better able to retain individuals (Aim 1), then our primary interest will be to ensure that adherence is as good or better in the intervention group. Based on GHESKIO data, we estimate that 10% of participants in the standard group will be non-adherent to ART, as defined by receiving ≤95% of prescribed ART medications in the first six months of ART. For a one-sided hypothesis test at the 0.05 significance level, a sample of 177 individuals in both arms is sufficient for 80% power to detect an increase in the proportion of non-adherent participants from 10% to 20%. Inflating our sample size to account for the estimated 24% of participants in the standard and 12% in the same-day ART group with attrition prior to completion of six months of ART (includes attrition prior to ART initiation and in the first six months of ART) yields an adjusted sample size of 233 participants in the standard and 202 in the same-day ART group. Therefore the 349 individuals randomized to each group (for Aim 1) will be sufficient to test this non-inferiority hypothesis.

For the sub-study, we calculated the sample size using the two-sided comparison of proportions. We anticipate a decrease in negative outcomes with same-day ART, but since this is a new approach, we will look for both increases and decreases in negative outcomes. We estimate that 20% of participants in the standard pre-ART group and 5% in the same-day ART group will have died or become lost to care at 6 months after study enrollment. There will be 80% power at a 0.05 significance level to detect this difference with 88 participants per arm, or 176 participants in the total cohort.

Patients in the main study and the sub-study will be excluded if they transfer. The sample size for the main study and the sub-study is 874, excluding transfers. We anticipate a transfer rate of 7%, and there will increase enrollment to 938 participants.

## 7.3 Analyses

Data will be exported into SAS software for analysis. All analyses will be conducted with an “intention-to-treat” approach.

*Aim 1 (Primary Aim):* We will compare the proportion of participants who are alive and in care with an HIV-1 RNA level <50 copies/ml in the standard and same-day ART groups at 12 months after HIV testing (binary outcome) using the two-sample test of proportions. The following variables are associated with mortality in patients on ART: age, gender, active TB, baseline body weight and CD4 cell count. We assume that these predictors of mortality will be similar between the two groups through the process of randomization, but we will explore any residual confounding for our primary outcome using logistic regression, with calculation of odds ratios and 95% confidence intervals. For the sub-study, we will compare the proportion of patients who are alive and in care at 6 months after study enrollment.

*Aim 2 (Secondary Aim):* We will use two methods to compare six-month ART adherence between the standard and the same-day ART groups. First, we will measure adherence using the medication possession ratio. Six-month adherence will be measured by adding up the number of pills dispensed in the first 180 days, and dividing this by the number of pills that should have been dispensed in 180 days with perfect adherence. We will compare the proportion of participants in each group who received >95% of antiretroviral medications in the first 180 days of ART using the two-sample test of proportions. We will also use the two-sample test of proportions to compare the proportion of participants in each group who report perfect adherence (missing 0 doses in the prior three days) on the questionnaire of self-reported three-day adherence described above.

*Aim 3:* We will compare the cost and cost-effectiveness of standard and same-day ART, where cost is measured by the mean treatment cost and effectiveness is measured by being alive and in care with a plasma HIV-1 RNA level <50 copies/ml at 12 months after HIV testing. We will conduct these analyses from the health system perspective (base case analysis) and the societal perspective. In conducting the analyses from the health system perspective, we will include the utilization and cost of all health services provided to each participant at GHESKIO and other outpatient and inpatient providers for the first 12 months after HIV testing. In conducting the analyses from the societal perspective, we will also include cost estimates for participant and family member time and transportation for medical visits.

First, we will conduct a cost analysis to measure the treatment cost per participant enrolled in the study from the health system perspective. We will use trial data to measure the utilization of ART and other medications, laboratory tests, radiographic studies, procedures, hospital services, labor, and telephone/transportation subsidies provided to participants, as we have done in the CIPRA cost-effectiveness evaluation described above, and in prior studies. We will obtain medication, laboratory, radiograph, labor and overhead costs and phone/transportation subsidies from the GHESKIO clinic, and hospital and procedure costs from local providers. Each of the resources used by each participant during the study period will be multiplied by the unit cost of that item and then summed to determine total costs for the study group. To calculate the mean cost of treatment per participant enrolled in the study, we will divide the relevant total cost of treatment for each group by the number of participants randomized to that group. Differences between arms in median and mean costs will be compared using the Wilcoxon rank-sum test and non-parametric bootstrap methods to account for potential skewness in cost data.

Next, we will conduct a cost-effectiveness analysis from the health system perspective, calculating the mean treatment cost per participant who is alive, in care and responding to ART (plasma HIV-1 RNA level <50 copies at 12 months after HIV testing). To do this, we will classify participants as (1) Alive, in care, and responding to ART (plasma HIV-1 RNA level <50 copies at 12 months after HIV testing); (2) Alive and in care but not responding to ART (plasma HIV-1 RNA level ≥50 copies at 12 months after HIV testing); or (3) No longer in care (did not attend the 12-month study visit). We will calculate the mean cost per participant who is alive, in care, and responding to ART by dividing the relevant total cost of treatment for each group by the number of participants who are alive, in care, and responding to ART. Finally, we will determine the incremental cost-effectiveness ratio (ICER), which is the ratio of the change in costs to the change in effects. The cost-effectiveness (CE) plane will be constructed with bootstrap samples and 95% CI will be computed. CE acceptability curves will also be drawn in order to understand the probability that same-day ART is more cost-effective for different willingness to pay thresholds. Sensitivity analyses based on the different cost settings (e.g., different cost assumptions, important subgroup analyses) will be performed, from which the most and least favorable scenarios among various real world settings may be investigated and elucidated. We will repeat the cost and cost-effectiveness analyses from the societal perspective. This will include the *addition* of cost estimates for participant and family member time spent on medical visits, and the estimated actual cost for transportation to medical visits based on the location of each participant’s residence.

# 8.0 DATA COLLECTION, MONITORING AND ADVERSE EVENT REPORTING

8.1 Records to Be Kept

Demographic, clinical, and laboratory information for all participants will be entered into the GHESKIO electronic medical record, as for non-research patients. All laboratory tests will be performed for routine clinical care except HIV-1 viral load testing, which will be done at six months after ART initiation and 12 months after study enrollment. Currently at GHESKIO, viral load testing is done only for patients with risk factors for treatment failure, due to the high cost of these tests. Viral load test results will be shared with the study physician to improve the clinical care of the subjects. Study questionnaires include the HIV medication readiness questionnaire and an adherence questionnaire that has been widely used for this purpose in studies in the U.S. and internationally, and has been culturally-adapted and translated into Creole and used in multiple NIH-funded GHESKIO studies. Both surveys will be administered in Creole; the HIV medication readiness questionnaire will be administered at screening, and the adherence questionnaire will be administered six months after ART initiation.

We will collect all clinical and laboratory data during the study period for the cost-effectiveness analysis. Study data will be extracted from the GHESKIO electronic medical record into Microsoft Excel and Access databases, which will be password protected. No names will be extracted from the electronic medical record. Subjects will be identified only by a unique study patient identification number on study questionnaires and documents. The list linking patient names and study numbers will be kept in a locked cabinet, which is accessible only to the PI and the GHESKIO Director. An IT programmer and a data manager will maintain this database, under the supervision of the research team.

If the participant provides a separate informed consent signature for audiotaping social worker counseling sessions, which will be conducted for quality control purposes, then all scheduled social worker sessions will be audiotaped. Audiotapes will be identified only by study ID number, and they will not be copied. They will be kept at GHESKIO in a locked cabinet. Access will be given only to the study PI and the GHESKIO Director.

8.2 Data Management and Security

GHESKIO has a central database system that manages information for over 250,000 patients a year. This system is used for multiple NIH-funded studies, including AIDS Clinical Trials Group and HIV Vaccine Trials Network studies, in addition to study-specific databases. To provide database backup, a full backup of the databases is automatically completed every night. The backup files are copied with the Microsoft ROBOCOPY tool to a SAN or External Hard Drive. That copy is overwritten on the same date each month. A differential backup of the databases is also performed every night and the backup files are copied every night. Every 30 minutes, a transaction log backup is also automatically done.

The electronic medical record was created by the GHESKIO IT team, with data stored in Microsoft SQL Server. All service patients are assigned a unique identifying number on their first visit to GHESKIO. Basic demographic data, the patient’s address, cell phone number, and a contact person are recorded at the first visit. All subsequent clinic visits and laboratory data are entered in real time into the electronic medical record by clinicians and laboratory staff. Microsoft Active Directory is used to restrict access to the clinical data system, and access is protected by password.

No research data will be emailed from GHESKIO to other sites (including Partners). No research data will be kept on USB drives. All study data that is removed from the secure GHESKIO system will be de-identified and maintained on computers that are protected by password, and by Safeboot.

8.3 Monitoring and Quality Assurance

GHESKIO is an international site of the NIH/NIAID Clinical Trials network. We will use similar methods that we use for NIH/NIAID AIDS Clinical Trials Group studies to monitor and assure the validity and integrity of the data and adherence to the IRB-approved protocol. At GHESKIO, Quality Control is done on 100% of all source documents and case report forms in real time on a daily basis in three separate areas for complete assessment: clinic, laboratory, and data management. Quality Assurance (QA) is done on an ongoing basis. QA is done on: 1) Twenty percent of research records; 2) Randomly chosen records; 3) All new subject screening and enrollment visits; 4) All records completed by new staff; and 5) All IRB documents. All trends are communicated to staff at weekly staff meetings. SOPs are written to resolve issues and detail procedures to prevent future errors.

8.4 Adverse Event Reporting

The following plans will be executed if a potential adverse effect of our intervention occurs:

1. The adverse event will be documented in the medical record, and in an appropriate case report form. A case report form will be filled out for all NIH Division of AIDS (DAIDS) grade 3 (severe) or grade 4 (life-threatening) events. The DAIDS Table for Grading the Severity of Adult and Pediatric Adverse Events, Version 1.0, December 2004 (Clarification, August 2009), will be used to grade adverse events. It is available on the DAIDS Web site at <http://rsc.tech-res.com/safetyandpharmacovigilance/>.
2. Patients with adverse events from ART will be managed according to standard GHESKIO protocols. A report form will also be filled out for any complaint regarding loss of confidentiality.
3. The PI and the GHESKIO Director will be notified if any adverse event occurs, including a DAIDS stage 3 or 4 event or complaint regarding loss of confidentiality.
4. For possible medical adverse events, participants will be evaluated within 24 hours (if non-life threatening) or within 2 hours (if life-threatening) of diagnosis of a possible adverse event. The study staff will assist patients in receiving immediate medical attention.
5. Each month, a report of adverse events by study group will be sent to the IRBs at all participating institutions, and a representative of the funding source. This will include all DAIDS grade 3 and 4 events, and all complaints regarding loss of confidentiality.

8.5 Institutional Review Board Review and Informed Consent

All human subjects research will be performed at the GHESKIO Center in Port-au-Prince, Haiti. All GHESKIO studies are approved by the GHESKIO IRB, as well as the IRBs of all collaborating organizations. The proposed research will be integrated into the overall GHESKIO HIV/AIDS treatment program, so Dr. Jean William Pape will have oversight for all activities in his capacity as Director of GHESKIO. The GHESKIO IRB, and the IRBs at all participating institutions, will review all relevant study proposals, SOPs, and documents. Data management for interventional clinical research trials at GHESKIO has been established during nearly 30 years of conducting clinical research in Haiti. All clinical information for the study will be maintained in the GHESKIO electronic medical record, which is password-protected.

Informed consent will be obtained in order to collect any information and material on study participants. Research material obtained from human subjects will be in the form of data, collected through questionnaires and medical chart review. All laboratory tests will be performed for routine clinical care except HIV-1 viral load testing, which will be done at 6 months after ART initiation and 12 months after study enrollment. Currently at GHESKIO, viral load testing is done only for patients with risk factors for treatment failure, due to the high cost of these tests. Viral load test results will be used to provide objective evidence of patient outcomes in the study. These viral load test results will also be used to improve the clinical care of the patients, and will be shared with the study physician. If participants sign the informed consent separately to provide permission for audiotaping of social worker visits, then all scheduled social worker visits will be audiotaped for quality control.

8.6 Subject Confidentiality

GHESKIO has longstanding procedures in place to protect subject confidentiality; all of these procedures will be followed for this study as well. All study staff will be trained in the conduct of ethical human subjects research and will sign a confidentiality pledge. We will prevent linkage of study information to an identifiable participant. Each participant will be assigned a unique ID number, which will be used for all study forms. All paper forms will be kept in a locked cabinet and electronic data will be housed in a safeguarded database, to which only study collaborators will have access. Data in this database will be stored under a coded ID number, which corresponds to each subject. The data which links the individual’s name to the coded ID number will be stored in a separate locked cabinet, will not be entered into a computer database, and will only be accessible to the study PI and the GHESKIO Director. Any identifying data will be removed from the database prior to performing study analysis.

8.7 Data Safety Monitoring Board

A Data Safety Monitoring Board (DSMB) has been designated to oversee the safety and effectiveness of the study. This committee will be chaired by Dr. Carlos del Rio, Professor and Chair of the Hubert Department of Global Health at the Rollins School of Public Health and Professor of Medicine in the Division of Infectious Diseases at the Emory University School of Medicine. He is also Co-director for the Clinical Science and International Research Core of the Emory Center for AIDS Research, and is highly experienced in conducting HIV research in resource-poor settings. The statistical expert on the DSMB will be Dr. Lawrence Moulton, Professor in the Department of International Health at The Johns Hopkins Bloomberg School of Public Health, with a joint appointment in the Department of Biostatistics. Professor Moulton is very experienced in conducting randomized trials. The DSMB will also include Dr. Kenneth Mayer, Director of HIV Prevention at Beth Israel Deaconess Hospital, Medical Research Director of the Fenway Institute, Visiting Professor at Harvard Medical School, and Adjunct Professor of Medicine and Epidemiology at Brown Medical School. Dr. Mayer has a very long history of conducting HIV prevention and treatment studies.

After six months of accrual, we will conduct an interim analysis on the rates of loss to follow-up (LTFU) at two months, using a one-sided Fisher exact test of futility with p-value cut-off of 0.05. We anticipate 246 patients (123 per arm) will have at least two months of potential follow-up six months after study initiation. If the true two-month LTFU rates are 18% in the control arm (15% pre-ART and 3% from the first month post-ART), we would have an 80% chance of stopping accrual at the interim if the LTFU rates were 15% higher (33% LTFU) in the intervention arm.  The final analysis will be based on a different endpoint, the 12-month rates of "LTFU or detectable viremia or death" and will use a two-sided 0.05 test.

We decided not to adjust the two-sided nominal p-value for the final analysis for two reasons. First, even if both analyses used the same endpoint and a two-sided test, using an O'Brien Fleming effectiveness boundary combined with a futility boundary would result in a nominal p-value for the final analysis of 0.0498. Also, the endpoint used in the interim analysis is not the same as the endpoint used in the final analysis. While the two endpoints may be related, we do not know the mathematical form of the relationship, and in analogy with clinical trials that standardly do not adjust survival tests for prior interim analyses of time to failure because of just such an unknown relationship, we will not adjust the final test in our study.

A design review meeting will be held with the DSMB prior to implementation of the study. Thereafter, the study will be presented for review by the DSMB at each of their semi-annual meetings. The DSMB will review data related to recruitment, randomization, compliance, and subject safety. Accrual, study conduct (including timeliness and completeness of data collection, fulfillment of eligibility criteria, protocol adherence, trial operating procedures, and form completion), adverse events, and deaths will be monitored. The DSMB will identify needs for additional data relevant to safety issues, and make recommendations on the continuation of the study, with regard to recruitment, compliance with protocols, and safety issues. The outcome of each DSMB review will be summarized in a letter to the IRBs of all participating institutions.

8.8 Study Discontinuation

Participants will not be removed from the study unless they request to withdraw from the study, or the study is stopped or cancelled early. Participants that withdraw from the study will be assured that they will receive HIV care as usual at GHESKIO, and that all services will be provided free of charge, as for all GHESKIO patients. After completion of the study, participants will be able to continue the same ART regimen free of charge at GHESKIO. For the sub-study, patients who are randomized to the same-day ART group who either withdraw or complete the study will be offered ongoing ART free of charge. Patients who are randomized to the standard pre-ART group will continue pre-ART care until they meet WHO guidelines for ART initiation.

9.0 RISKS AND DISCOMFORTS

9.1 Complications of Surgical and Non-Surgical procedures: None

9.2 Drug Side Effects and Toxicities:

Participants in this study will receive the same antiretroviral therapy and other medications that they would receive in standard of care at GHESKIO.

- 1. Device Complications/Malfunctions: None

9.4 Psychosocial (Non-Medical Risks):

Upon diagnosis of HIV, individuals are vulnerable to stigma associated with this disease in the community. It is possible that participation in this study could magnify this stigma by drawing attention to the subject’s medical status because of phone calls or home visits for missed visits throughout the study period. Though participants in the same-day ART group will have comprehensive ART literacy and adherence counseling, they could feel psychological stress or discomfort from starting ART on the day of HIV testing. It is possible that some patients will have a difficult time learning their HIV status and starting same-day ART. The same-day ART group will receive support in coping with the diagnosis, disclosing their HIV status, and adhering to ART through the five physician, pharmacist, and social worker visits within the first month of ART initiation (this includes physician, pharmacist and social worker visits three days after ART initiation). This high frequency of early physician visits will also facilitate immediate and appropriate management of adverse events. All study participants (both groups) will also be given access to cell phones and phone cards if they do not have them. Cell phones can be easily re-charged in Haiti, even for participants that do not have electricity in their homes. Like all ART patients at GHESKIO, participants in both groups will also have access to a physician on call 24 hours/day to provide immediate services for those in need, and if they miss a visit, they will be phoned by a community health worker (CHW); those who cannot be reached by phone will receive a home visit by a CHW. All participants will receive adherence counseling prior to ART initiation. Participants in either study group who are determined to be non-compliant will receive additional counseling sessions, as is standard of care at GHESKIO.

Participants in the study could also feel stress from completing the adherence self-report. We will implement additional measures to reduce the impact of psychological distress for the participants in this study. First, all study personnel will be trained to treat participants with respect and dignity, and will only approach participants in areas and at times that the participant finds acceptable. Second, study personnel, including the CHWs, will also receive training on stigma and psychosocial implications of HIV prior to initiating work in this study. The CHWs will not identify themselves as being from GHESKIO when they call or make a home visit, or when they talk to another member of the participant’s household.

An additional concern is the possibility of loss of confidentiality due to participation in this study. All study personnel will be certified in Human Subjects Research, and they will receive additional training in confidentiality prior to working with patients. We will ensure that all participants have access to the PI and the study staff for any events concerning stigma, violation of confidentiality, or unwanted disclosure that may occur as a result of being in the study. Any complaint will be communicated to the PI and the GHESKIO Director, and reported as an adverse event related to the study. We note that in nearly 30 years of conducting HIV-related research in Haiti, GHESKIO has never had any problems regarding participant confidentiality.

10.0 POTENTIAL BENEFITS

10.1 Potential Benefits to Participating Individuals

The potential benefit from participating in this study is that the participant may improve his/her health by being less likely to be lost to care prior to ART initiation, if he/she responds positively to the same-day ART intervention. In addition, all participants, regardless of treatment assignment, may benefit from virologic monitoring at six months post-ART initiation and at 12 months after study enrollment, and from closer care (proximity to the study team). Participants may also gain an increased awareness of HIV; they may also sense an increased level of concern regarding HIV and social support among the study personnel and the HIV providers. Patients in the sub-study who are randomized to same-day ART may benefit from the earlier initiation of ART; we note that it is standard of care for all HIV-infected patients in the United States to receive ART.

- 1. Potential Benefits to Society

The interventions in the study may benefit not only the study subjects, but other HIV-positive individuals in Haiti and other resource-poor settings. All sites that have reported on pre-ART outcomes have reported high levels of pre-ART attrition. If the proposed research demonstrates that it is safe and effective to provide same-day ART, then other programs around the world are likely to consider the possibility that they could minimize their pre-ART requirements for ART literacy and adherence counseling visits and medical evaluations for OIs. Even though GHESKIO conducts research studies, the standard of care at GHESKIO is similar to other HIV treatment programs in resource-poor settings. GHESKIO faces the same budget and infrastructure challenges and uses similar treatment protocols as other resource-poor settings.

11.0 STUDY REMUNERATION

All care at GHESKIO is provided free of charge to patients, regardless of whether they are participating in a study. GHESKIO is the largest provider of HIV services in the Caribbean. Study participants will receive $US 2.50 for every study visit as a subsidy to cover the cost of their transportation to clinic.

12.0 PUBLICATION OF RESEARCH FINDINGS

Publication of the results of this trial will be governed by NIH policies.

13.0 REFERENCES

1. Mulissa Z, Jerene D, Lindtjorn B. Patients present earlier and survival has improved, but pre-ART attrition is high in a six-year HIV cohort data from Ethiopia. PloS one 2010;5:e13268.

2. Amuron B, Namara G, Birungi J, et al. Mortality and loss-to-follow-up during the pre-treatment period in an antiretroviral therapy programme under normal health service conditions in Uganda. BMC public health 2009;9:290.

3. Toure S, Kouadio B, Seyler C, et al. Rapid scaling-up of antiretroviral therapy in 10,000 adults in Cote d'Ivoire: 2-year outcomes and determinants. AIDS 2008;22:873-82.

4. Micek MA, Gimbel-Sherr K, Baptista AJ, et al. Loss to follow-up of adults in public HIV care systems in central Mozambique: identifying obstacles to treatment. J Acquir Immune Defic Syndr 2009;52:397-405.

5. Bassett IV, Regan S, Chetty S, et al. Who starts antiretroviral therapy in Durban, South Africa?... not everyone who should. AIDS 2010;24 Suppl 1:S37-44.

6. Larson BA, Brennan A, McNamara L, et al. Early loss to follow up after enrolment in pre-ART care at a large public clinic in Johannesburg, South Africa. Tropical medicine & international health : TM & IH 2010;15 Suppl 1:43-7.

7. Kranzer K, Zeinecker J, Ginsberg P, et al. Linkage to HIV care and antiretroviral therapy in Cape Town, South Africa. PloS one 2010;5:e13801.

8. Bassett IV, Wang B, Chetty S, et al. Loss to care and death before antiretroviral therapy in Durban, South Africa. J Acquir Immune Defic Syndr 2009;51:135-9.

9. Noel E, Esperance M, McLaughlin M, et al. Attrition from HIV Testing to Antiretroviral Therapy Initiation among Patients Newly Diagnosed with HIV in Haiti. J Acquir Immune Defic Syndr 2012.

10. Losina E, Bassett IV, Giddy J, et al. The "ART" of linkage: pre-treatment loss to care after HIV diagnosis at two PEPFAR sites in Durban, South Africa. PloS one 2010;5:e9538.

11. Larson BA, Brennan A, McNamara L, et al. Lost opportunities to complete CD4+ lymphocyte testing among patients who tested positive for HIV in South Africa. Bulletin of the World Health Organization 2010;88:675-80.

12. Ingle SM, May M, Uebel K, et al. Outcomes in patients waiting for antiretroviral treatment in the Free State Province, South Africa: prospective linkage study. AIDS 2010;24:2717-25.

13. Tayler-Smith K, Zachariah R, Massaquoi M, et al. Unacceptable attrition among WHO stages 1 and 2 patients in a hospital-based setting in rural Malawi: can we retain such patients within the general health system? Transactions of the Royal Society of Tropical Medicine and Hygiene 2010;104:313-9.

14. Rosen S, Fox MP. Retention in HIV care between testing and treatment in sub-Saharan Africa: a systematic review. PLoS medicine 2011;8:e1001056.

15. McGuire M, Munyenyembe T, Szumilin E, et al. Vital status of pre-ART and ART patients defaulting from care in rural Malawi. Tropical medicine & international health : TM & IH 2010;15 Suppl 1:55-62.

16. McGrath N, Glynn JR, Saul J, et al. What happens to ART-eligible patients who do not start ART? Dropout between screening and ART initiation: a cohort study in Karonga, Malawi. BMC public health 2010;10:601.

17. Lessells RJ, Mutevedzi PC, Cooke GS, Newell ML. Retention in HIV care for individuals not yet eligible for antiretroviral therapy: rural KwaZulu-Natal, South Africa. J Acquir Immune Defic Syndr 2011;56:e79-86.

18. World Health Organization (2010) Antiretroviral Therapy for HIV Infection in Adults and Adolescents Recommendations for a Public Health Approach 2010 Revision. Geneva, Switzerland: World Health Organization. Accessed May 1, 2013 at: <http://whqlibdoc.who.int/publications/2010/9789241599764_eng.pdf>.

19. Mills EJ, Nachega JB, Buchan I, et al. Adherence to antiretroviral therapy in sub-Saharan Africa and North America: a meta-analysis. JAMA : the journal of the American Medical Association 2006;296:679-90.

20. Orrell C. Antiretroviral adherence in a resource-poor setting. Current HIV/AIDS reports 2005;2:171-6.

21. Gardner EM, McLees MP, Steiner JF, Del Rio C, Burman WJ. The spectrum of engagement in HIV care and its relevance to test-and-treat strategies for prevention of HIV infection. Clinical infectious diseases : an official publication of the Infectious Diseases Society of America 2011;52:793-800.

22. Mtapuri-Zinyowera S, Chideme M, Mangwanya D, et al. Evaluation of the PIMA point-of-care CD4 analyzer in VCT clinics in Zimbabwe. J Acquir Immune Defic Syndr 2010;55:1-7.

23. Jani IV, Sitoe NE, Chongo PL, et al. Accurate CD4 T-cell enumeration and antiretroviral drug toxicity monitoring in primary healthcare clinics using point-of-care testing. AIDS 2011;25:807-12.

24. Glencross DK, Coetzee LM, Faal M, et al. Performance evaluation of the Pima point-of-care CD4 analyser using capillary blood sampling in field tests in South Africa. Journal of the International AIDS Society 2012;15:3.

25. Chalker JC, Andualem T, Gitau LN, et al. Measuring adherence to antiretroviral treatment in resource-poor settings: the feasibility of collecting routine data for key indicators. BMC health services research 2010;10:43.

26. Bell DJ, Kapitao Y, Sikwese R, van Oosterhout JJ, Lalloo DG. Adherence to antiretroviral therapy in patients receiving free treatment from a government hospital in Blantyre, Malawi. J Acquir Immune Defic Syndr 2007;45:560-3.

27. Mills EJ, Nachega JB, Bangsberg DR, et al. Adherence to HAART: a systematic review of developed and developing nation patient-reported barriers and facilitators. PLoS medicine 2006;3:e438.

28. Ross-Degnan D, Pierre-Jacques M, Zhang F, et al. Measuring adherence to antiretroviral treatment in resource-poor settings: the clinical validity of key indicators. BMC health services research 2010;10:42.

29. Nachega JB, Hislop M, Dowdy DW, et al. Adherence to highly active antiretroviral therapy assessed by pharmacy claims predicts survival in HIV-infected South African adults. J Acquir Immune Defic Syndr 2006;43:78-84.

30. Bisson GP, Gross R, Bellamy S, et al. Pharmacy refill adherence compared with CD4 count changes for monitoring HIV-infected adults on antiretroviral therapy. PLoS medicine 2008;5:e109.

31. Cohen MS, Chen YQ, McCauley M, et al. Prevention of HIV-1 infection with early antiretroviral therapy. The New England journal of medicine 2011;365:493-505.

32. Granich RM, Gilks CF, Dye C, De Cock KM, Williams BG. Universal voluntary HIV testing with immediate antiretroviral therapy as a strategy for elimination of HIV transmission: a mathematical model. Lancet 2009;373:48-57.

33. Okulicz JF, Le TD, Agan BK, et al. Influence of the Timing of Antiretroviral Therapy on the Potential for Normalization of Immune Status in Human Immunodeficiency Virus 1-Infected Individuals. JAMA internal medicine 2014.

34. Panel on Antiretroviral Guidelines for Adults and Adolescents. Guidelines for the use of

antiretroviral agents in HIV-1-infected adults and adolescents. Department of Health and

Human Services. Accessed December 1, 2014 at <http://aidsinfo.nih.gov/ContentFiles/Adultand>

AdolescentGL.pdf.

35. Antiretroviral Therapy for HIV Infection in Adults and Adolescents. Recommendations for a Public Health Approahc, 2010 Revision, World Health Organization, Geneva, Switzerland, 2010. Access March 6, 2013 at: <http://whqlibdoc.who.int/publications/2010/9789241599764_eng.pdf>.

36. Bassett IV, Sax PE. Untreated HIV: harmful even at high CD4 cell counts. Lancet 2010;376:306-8.

37. Lodwick RK, Sabin CA, Porter K, et al. Death rates in HIV-positive antiretroviral-naive patients with CD4 count greater than 350 cells per microL in Europe and North America: a pooled cohort observational study. Lancet 2010;376:340-5.

38. Gabillard D, Lewden C, Ndoye I, et al. Mortality, AIDS-morbidity, and loss to follow-up by current CD4 cell count among HIV-1-infected adults receiving antiretroviral therapy in Africa and Asia: data from the ANRS 12222 collaboration. J Acquir Immune Defic Syndr 2013;62:555-61.

39. Olubajo B, Mitchell-Fearon K, Ogunmoroti O. A Comparative Systematic Review of the Optimal CD4 Cell Count Threshold for HIV Treatment Initiation. Interdisciplinary perspectives on infectious diseases 2014;2014:625670.

40. Wood R, Lawn SD. Antiretroviral treatment as prevention: impact of the 'test and treat' strategy on the tuberculosis epidemic. Current HIV research 2011;9:383-92.

41. Mocroft A, Furrer HJ, Miro JM, et al. The incidence of AIDS-defining illnesses at a current CD4 count >/= 200 cells/muL in the post-combination antiretroviral therapy era. Clinical infectious diseases : an official publication of the Infectious Diseases Society of America 2013;57:1038-47.

42. Kitahata MM, Gange SJ, Abraham AG, et al. Effect of early versus deferred antiretroviral therapy for HIV on survival. The New England journal of medicine 2009;360:1815-26.

43. Phillips AN, Gazzard B, Gilson R, et al. Rate of AIDS diseases or death in HIV-infected antiretroviral therapy-naive individuals with high CD4 cell count. AIDS 2007;21:1717-21.

44. Le T, Wright EJ, Smith DM, et al. Enhanced CD4+ T-cell recovery with earlier HIV-1 antiretroviral therapy. The New England journal of medicine 2013;368:218-30.

45. Consolidated Guidelines on the Use of Antiretroviral Drugs for Treating and Preventing HIV Infection. Recommendations for a Public Health Approach. June 2013. Geneva, Switzerland: World Health Organization, 2013.

46. Fast PE, Price MA, Rida WN, Kamali A, Karita E. WHO's new guidelines for antiretroviral treatment. Lancet 2013;382:1778-9.

47. Rouzier V, Farmer PE, Pape JW, et al. Factors impacting the provision of antiretroviral therapy to people living with HIV: the view from Haiti. Antiviral therapy 2014;19 Suppl 3:91-104.

48. Severe P, Juste MA, Ambroise A, et al. Early versus standard antiretroviral therapy for HIV-infected adults in Haiti. The New England journal of medicine 2010;363:257-65.

49. Koenig SP, Bang H, Severe P, et al. Cost-effectiveness of early versus standard antiretroviral therapy in HIV-infected adults in Haiti. PLoS medicine 2011;8:e1001095.

50. Koenig SP, Riviere C, Leger P, et al. High mortality among patients with AIDS who received a diagnosis of tuberculosis in the first 3 months of antiretroviral therapy. Clinical infectious diseases : an official publication of the Infectious Diseases Society of America 2009;48:829-31.

51. Koenig SP, Rodriguez LA, Bartholomew C, et al. Long-Term Antiretroviral Treatment Outcomes in Seven Countries in the Caribbean. J Acquir Immune Defic Syndr;59:e60-e71.

52. Severe P, Juste MA, Ambroise A, et al. Early versus standard antiretroviral therapy for HIV-infected adults in Haiti. N Engl J Med;363:257-65.

SAMPLE INFORMED CONSENT – *FOR PATIENTS WITH CD4 COUNT ≤500 CELLS/mm^3^*

INTRODUCTION

You are being asked to take part in this research study because you are infected with HIV (the virus that causes AIDS). This study is sponsored by the National Institutes of Health (NIH). GHESKIO is the only site for the study. The doctors in charge of this study at GHESKIO are Dr. Patrice Severe, Dr. Jean William Pape, and Dr. Serena Koenig. Before you decide if you want to be a part of this study, we want you to know about the study.

This is a consent form. It gives you information about this study. The study staff will talk with you about this information. You are free to ask questions about this study at any time. If you agree to take part in this study, you will be asked to sign this consent form. You will get a copy to keep.

WHY IS THIS STUDY BEING DONE?

International guidelines (and Haitian National Guidelines) recommend that all patients with HIV and a CD4 cell count (a type of blood cell) that is lower than 500 cells/mm^3^ should start treatment with antiretroviral therapy (ART). The current standard of practice in Haiti and other resource-poor countries is for patients to attend multiple visits for counseling and baseline testing prior to starting ART. Many patients are lost to follow-up during these visits, and they never start ART. Studies that have evaluated reasons for this lost to follow-up prior to ART have found that multiple visits are a barrier for patients.

This research is being done to evaluate whether starting ART on the day of HIV testing would improve retention in care and patient outcomes, compared with standard of care (three visits prior to ART initiation). You are being asked to take part in this research study because you are infected with HIV, the virus that causes AIDS, and you qualify for ART because your CD4 cell count is lower than 500 cells/mm^3^.

WHAT DO I HAVE TO DO IF I AM IN THIS STUDY?

If you decide to join this study, you will need to be seen in the clinic about 16 times during the year of the study. The evaluations required at most visits will take about one hour to complete, although you may need to be at the clinic longer than this.

If you do not join the study

If you decide not to take part in this study, you will be able to receive ART and other HIV services at GHESKIO. All treatment at GHESKIO is free of charge to everyone, regardless of whether or not you participate in a research study.

If you enter the study

At the study entry visit, you will be assigned to one of these two treatment groups:

Study Entry

Standard Antiretroviral Therapy

Same-Day Antiretroviral Therapy

Because your assignment is random, like the flip of a coin, you will have an equal chance of being in either group. You will not be able to choose your group, but you and the study doctor, as well as the study staff, will know which group you are in.

Standard Group: If you are assigned to the standard group, you will receive treatment that is very similar to the treatment provided to other patients at GHESKIO that are not participating in a research study. You will have three clinic visits prior to starting ART. During each of these visits, you will meet with a physician and a social worker. During the physician visit, you will be evaluated for other illnesses with blood tests (kidney tests, liver tests, complete blood count, and other tests your doctor thinks are indicated). The social worker will explain the importance of taking your medications every day, and help you to come up with a plan to take your treatment as prescribed.

After this, you will start ART. Prior to starting ART, you will meet with a pharmacist, who will counsel you about the importance of taking your medications as prescribed, and will dispense your medications to you. After this, you will have another physician visit in two weeks, and then have a monthly physician visit throughout the year of the study. At every visit you will receive additional ART, and additional counseling by the pharmacist who will dispense your medications to you. Every three months, you will have a social worker visit; during these visits, the social worker will talk to you about how you are feeling, and help you to adhere to your treatment. If you miss a visit, you will be phoned by a GHESKIO field worker. If you do not answer the phone, the field worker will visit you at your home to determine how they can help you return to clinic.

Same-Day ART Group: If you are assigned to the same-day ART group, you will receive additional visits today from the social worker, the physician, and the pharmacist. They will explain the purpose of taking ART, and the importance of taking your medications every day. The physician will also evaluate you for other medical illnesses with blood tests (kidney tests, liver tests, complete blood count, and other tests your doctor thinks are indicated). After this, you will start ART (later today). You will receive the same ART medications that you would have received if you had been randomized to the standard group.

You will have a follow-up appointment in three days, which will include a medical evaluation by the study physician and ART adherence counseling from a social worker. You will then have weekly visits with the physician and social worker for the three subsequent weeks. Pharmacists will provide you with adherence counseling at every ART visit. After these initial visits, you will have monthly visits with a physician throughout the year of the study. At every visit you will receive additional ART, and additional counseling by the pharmacist who will dispense your medications to you. Every three months, you will have a social worker visit; during these visits, the social worker will talk to you about how you are feeling, and help you to adhere to your treatment. If you miss a visit, you will be phoned by a GHESKIO field worker. If you do not answer the phone, the field worker will visit you at your home to determine how they can help you return to clinic.

Regardless of the treatment group you are assigned to, you will receive the same ART treatment as for non-research patients at GHESKIO. You will also receive the same laboratory tests as non-research patients at GHESKIO receive, except you will receive an HIV viral load test at 6 months at 12 months after study enrollment. This test is recommended by the World Health Organization for all patients on ART, but due to the high cost of the test, it is not done routinely in Haiti.

If you choose to participate in this study, no specimens will be collected for future research, or for sending to collaborators outside of Haiti. If you choose to participate in this study, we will ask you to complete three questionnaires about your medication adherence, and how the medications make you feel.

AUDIOTAPING OF SOCIAL WORKER COUNSELING SESSIONS

During the year of the study, you will have eight scheduled counseling sessions with a social worker. These sessions are very important, because the social worker will explain the importance of adhering to your medications, and help you to develop strategies to take your medications as directed. If you agree by signing the separate informed consent line below, then we will audiotape your social worker counseling sessions for quality control purposes. You will not be videotaped. Your audio recording will be identified only by a special study number, not by your name. The list linking your name and your study number will be kept in a locked cabinet at GHESKIO. Access to this list will be limited to key study personnel. Your audiotapes will never be made public, and they will not be copied. You can change your mind at any time, and stop having your social worker sessions audiotaped. You may also request that past audiotapes be destroyed; if requested by you at any time, we will destroy all of your audiotapes.

If you have had all your questions about audiotaping of the social worker sessions answered, and you agree to have your social worker sessions audiotaped, please sign your name below. At the end of the study, all audiotapes for all participants will be destroyed.

**Informed Consent for Audiotaping Only:**

____________________________ ___________________________________

Participant’s Name (print) Participant’s Signature and Date

____________________________ ___________________________________

Study Staff Conducting (print) Study Staff’s Signature and Date

HOW MANY PEOPLE WILL TAKE PART IN THIS STUDY?

About 698 people will take part in this study, after excluding participants who enroll and then transfer to another clinic. GHESKIO is the only site for this study.

HOW LONG WILL I BE IN THIS STUDY?

It will take you about one year to complete this study. During this time, we will ask you to make 16 study visits.

WHY WOULD THE DOCTOR TAKE ME OFF THIS STUDY EARLY?

The study doctor may need to take you off the study early without your permission if:

- The study is stopped or cancelled.
- A Data Safety Monitoring Board (DSMB) recommends that the study be stopped early. (A DSMB is an outside group of experts who monitor the study).

After you have completed the study, you will be able to continue the same ART at GHESKIO with the non-research patients. All care at GHESKIO is free of charge.

WHAT ARE THE RISKS OF THE STUDY?

You will not be receiving any procedures or HIV treatments in this study that are not provided as routine clinical care. The difference between the same-day ART group and the standard group is the timing of receiving ART (today or after three visits). If you are in the same-day ART group, you may feel psychological discomfort from starting treatment the same day you are diagnosed with HIV. It is also possible that participating in this study will make it difficult for you to keep your HIV status secret from people close to you. This may lead to unwelcome discussions about or reactions to your HIV status. Please talk with the study staff if you have any concerns in this regard. We will try to protect you against these risks by ensuring that you receive adequate counseling before you start same-day ART, and by ensuring that we carefully protect your confidentiality throughout the study.

ARE THERE BENEFITS TO TAKING PART IN THIS STUDY?

It is possible that you will not receive any benefit from participating in this study. Possible benefits to you will include the proximity to the study staff, phone calls and/or home visits if you miss a clinic visit, and testing of your HIV viral load at six and 12 months after study enrollment, which will help to determine if your ART is still effective, or if you have developed resistance to treatment, and require a change in ART. If you were to develop resistance to the first-line ART, then GHESKIO will provide you with second-line ART medications, as they do for non-research patients.

We expect that other HIV-infected patients in Haiti and other resource-poor settings will benefit from the findings of this study. In Haiti and other resource-poor countries, patients diagnosed with HIV are usually required to attend multiple clinic visits prior to starting ART. During this time, many patients are lost to care. If we demonstrate that same-day ART produces patient outcomes that are as good or better than standard approaches, it will suggest to other HIV programs in Haiti and other resource-poor countries that it is not necessary to require patients to attend multiple clinic visits prior to starting ART.

WHAT OTHER CHOICES DO I HAVE BESIDES THIS STUDY?

You do not need to participate in this study to receive HIV treatment at GHESKIO. All care at GHESKIO is provided free of charge to all patients. The HIV medications used in this study are the same ones that are used for non-research patients at GHESKIO.

WHAT SHOULD I DO IF I WANT TO STOP PARTICIPATING IN THIS STUDY?

You can decide to stop participating in this study at any time. If you decide to stop participating, speak with your study physician. You will be able to receive the same ART free of charge at GHESKIO if you choose to stop participating in this study.

# WILL I BE PAID TO TAKE PART IN THIS RESEARCH STUDY?

If you participate in this study, you will receive $US 2.50 per visit to cover the cost of your transportation for every study visit that you attend.

WHAT ABOUT CONFIDENTIALITY?

Efforts will be made to keep your personal information confidential. We cannot guarantee absolute confidentiality. Your personal information may be disclosed if required by law. Any publication of this study will not use your name or identify you personally. Your records may be reviewed by the NIH, and by the Institutional Review Boards at GHESKIO, Partners Healthcare, Harvard, Cornell University, and Florida International University. Your information may also be reviewed by study monitors, and by study staff.

WHAT ARE THE COSTS TO ME?

All outpatient HIV treatment at GHESKIO is provided free of charge to all patients. For patients that require hospitalization due to HIV or non-HIV-related illnesses, GHESKIO will attempt to find funding to cover the cost of hospitalization, as they do for all GHESKIO patients. This will depend on funds that are available to GHESKIO.

WHAT HAPPENS IF I AM INJURED?

Since you are receiving the same HIV medications that non-research patients receive, we do not expect any research-associated injuries from participation in this study. Injuries sometimes happen in research, even when no one is at fault. If you are injured as a result of being in this study, you will be given immediate treatment for your injuries. There are no plans to pay you or give you other compensation for an injury, should one occur. However, you will not be giving up any of your legal rights by signing this consent form.

WHAT ARE MY RIGHTS AS A RESEARCH PARTICIPANT?

Taking part in this study is completely voluntary. You may choose not to take part in this study or leave this study at any time. Your decision will not affect medical care you receive at this site.

We will tell you about new information from this or other studies that may affect your health, welfare, or willingness to stay in this study. If you want the results of the study, let the study staff know.

WHAT DO I DO IF I HAVE QUESTIONS OR PROBLEMS?

For questions or problems related to the study, you may call Dr. Jean William PAPE, who is the Director of GHESKIO and an investigator in the study, or Dr. Patrice SEVERE who is leading this study at GHESKIO, at the following telephone numbers: 2-222-0031/2-222-2241; or go to the GHESKIO Centers at the following Address: #31, Blvd. Harry Truman, Bicentenary, Port-au-Prince. You can also call Dr. Serena Koenig, who is the primary investigator in the study, either at 2-222-0031/2-222-2241 (GHESKIO numbers) or 617-413-4090 (US number). For questions about your rights as a participant in the study, you may call Dr. Rose-Irène VERDIER, who is the person in charge of having the rights of the participants in the study respected, at the following numbers: 2-222-0031/ 2-222-2241; or go to the GHESKIO Centers at the following Address: #31, Blvd. Harry Truman, Bicentenary, Port-au-Prince; or you may call Dr. Alix LASSEGUE, who is in charge of ethics at the GHESKIO Centers, at the following telephone number: 2-222-4743.

SIGNATURE PAGE

If you have read this consent form (or had it explained to you), all your questions have been answered, and you agree to take part in this study, please sign your name below.

____________________________ ___________________________________

Participant’s Name (print) Participant’s Signature and Date

____________________________ ___________________________________

Study Staff Conducting (print) Study Staff’s Signature and Date

_____________________________ ___________________________________

Name of Witness (print) Witness’s Signature and Date

SAMPLE INFORMED CONSENT

For the sub-study of protocol “Same-Day HIV Testing and Treatment Initiation to Improve Retention in Care” – this version of the informed consent form will be used for participants with CD4 count >500 cells/mm^3^.

INTRODUCTION

You are being asked to take part in this research study because you are infected with HIV (the virus that causes AIDS). This study is sponsored by the MAC AIDS Foundation. GHESKIO is the only site for the study. The doctor in charge of this study at GHESKIO is Dr. Jean William Pape. Before you decide if you want to be a part of this study, we want you to know about the study.

This is a consent form. It gives you information about this study. The study staff will talk with you about this information. You are free to ask questions about this study at any time. If you agree to take part in this study, you will be asked to sign this consent form. You will get a copy to keep.

WHY IS THIS STUDY BEING DONE?

United States guidelines recommend treatment with antiretroviral therapy (ART) for all patients who are HIV-infected, because it reduces the risk of HIV-related complications such as cardiac, kidney, and liver disease, and HIV-associated malignancies, and reduces the risk of HIV transmission to others. The World Health Organization and Haitian National Guidelines currently recommend that all patients with HIV and a CD4 cell count (a type of blood cell) that is lower than 500 cells/mm^3^ should start treatment with ART. The major reason for the difference in guidelines is due to lack of financial resources in Haiti and other resource-poor countries. Many patients who do not meet WHO eligibility criteria for ART initiation at HIV testing become lost to follow-up by the time they qualify for ART, which would generally occur within the subsequent one to two years.

This research is being done to evaluate whether retention in care would be higher if patients with early HIV disease (who do not yet qualify for ART by WHO guidelines) were treated with ART. You are being asked to take part in this research study because you are infected with HIV, the virus that causes AIDS, and you do not yet qualify for ART because your CD4 cell count is higher than 500 cells/mm^3^.

WHAT DO I HAVE TO DO IF I AM IN THIS STUDY?

If you decide to join this study, you will need to be seen in the clinic about 6 to 11 times during the 6-month study duration. The evaluations required at most visits will take about one hour to complete, although you may need to be at the clinic longer than this.

If you do not join the study

If you decide not to take part in this study, you will be able to receive HIV services at GHESKIO. All treatment at GHESKIO is free of charge to everyone, regardless of whether or not you participate in a research study.

If you enter the study

At the study entry visit, you will be assigned to one of these two treatment groups:

Study Entry

Standard Pre-ART Care

Same-Day Antiretroviral Therapy

Because your assignment is random, like the flip of a coin, you will have an equal chance of being in either group. You will not be able to choose your group, but you and the study doctor, as well as the study staff, will know which group you are in.

Standard Group: You will have monthly physician visits for the 3 first months, and then every other month physician visits. During the physician visit, you will be evaluated for other illnesses, and you will be provided with medications to protect you against infections such as tuberculosis. If you miss a visit, you will be phoned by a GHESKIO field worker. If you do not answer the phone, the field worker will visit you at your home to determine how they can help you return to clinic. After you complete the 6-month study, you will be followed in the GHESKIO pre-ART clinic, and you will continue to receive all care free of charge. GHESKIO clinicians will monitor you, and will recommend that you start ART when you meet the WHO eligibility criteria.

Same-Day ART Group: If you are assigned to the same-day ART group, you will receive additional visits today from the social worker, the physician, and the pharmacist. They will explain the purpose of taking ART, and the importance of taking your medications every day. The physician will also evaluate you for other medical illnesses with blood tests (kidney tests and other tests your doctor thinks are indicated). After this, you will start ART (later today). You will receive the same ART medications that other GHESKIO ART patients receive. You will have a follow-up appointment in three days, which will include a medical evaluation by the study physician and ART adherence counseling from a social worker. You will then have weekly physician visits for the three subsequent weeks, with two additional social worker visits. Pharmacists will provide you with adherence counseling at every ART visit. After the first month, you will have monthly clinic visits. After three months, participants who are asymptomatic with good adherence will be offered the opportunity to participate in the Rapid Pathway. Patients in the Rapid Pathway receive expedited care from a nurse, who will evaluate them for symptoms of illness, and dispense ART. If you miss a visit, you will be phoned by a GHESKIO field worker. If you do not answer the phone, the field worker will visit you at your home to determine how they can help you return to clinic. At the end of the 6-month study duration, you will receive an HIV viral load test. This test is recommended by the World Health Organization for all patients on ART, but due to the high cost of the test, it is not done routinely in Haiti. After you complete the 6-month study, you will be followed in the GHESKIO ART clinic, and continue to receive ART and all other GHESKIO HIV care free of charge.

If you choose to participate in this study, no specimens will be collected for future research, or for sending to collaborators outside of Haiti. If you choose to participate in this study, we will ask you to complete three questionnaires about how you feel about your HIV diagnosis. If you are randomized to the same-day ART group, we will ask you to complete a questionnaire about about your medication adherence, and how the medications make you feel.

If you choose not to participate in this study, we will ask you if we can use the information from your medical record to understand the acceptability of earlier ART for patients. We will also ask you if you are willing to tell us why you do not want to participate. We will only do this if you sign a consent form to give us permission.

HOW MANY PEOPLE WILL TAKE PART IN THIS STUDY?

About 176 people will take part in this study. GHESKIO is the only site for this study.

HOW LONG WILL I BE IN THIS STUDY?

It will take you 6 months to complete this study. During this time, we will ask you to make from 6 to 11 study visits.

WHY WOULD THE DOCTOR TAKE ME OFF THIS STUDY EARLY?

The study doctor may need to take you off the study early without your permission if the study is stopped or cancelled.

After you have completed the study, you will be able to continue the same treatment at GHESKIO with non-research patients. All care at GHESKIO is free of charge.

WHAT ARE THE RISKS OF THE STUDY?

If you are randomized to the standard group, you will not be receiving any procedures or HIV treatments in this study that are not provided as routine clinical care. If you are randomized to the same-day ART group, you may feel psychological discomfort from starting treatment the same day you receive your CD4 count results. If you initiate ART, you will need to take it every day, as prescribed by your doctor, to avoid developing resistance to the medications. You will be starting treatment approximately one to two years earlier than other patients in Haiti. It is also possible that participating in this study might make it difficult for you to keep your HIV status secret from people close to you. This may lead to unwelcome discussions about or reactions to your HIV status. Please talk with the study staff if you have any concerns in this regard. We will try to protect you against these risks by ensuring that you receive adequate counseling before you start same-day ART, and by ensuring that we carefully protect your confidentiality throughout the study. In particular, any GHESKIO staff that contact you (through home visits or by phone) will not identify themselves as working with GHESKIO.

ARE THERE BENEFITS TO TAKING PART IN THIS STUDY?

It is possible that you will not receive any benefit from participating in this study. Possible benefits to you will include the proximity to the study staff, phone calls and/or home visits if you miss a clinic visit. If you are randomized to the same-day ART group, you may benefit from earlier treatment with ART; you will also receive an HIV viral load test at 6 months after ART initiation, which will provide your doctor with information about how effectively the ART is working for you.

We expect that other HIV-infected patients in Haiti and other resource-poor settings will benefit from the findings of this study. This study will provide data on whether the use of ART for patients with early HIV disease, who do not yet meet WHO criteria for ART initiation, improves retention in care.

WHAT OTHER CHOICES DO I HAVE BESIDES THIS STUDY?

You do not need to participate in this study to receive HIV treatment at GHESKIO. All care at GHESKIO is provided free of charge to all patients. The HIV medications used in this study are the same ones that are used for non-research patients at GHESKIO.

WHAT SHOULD I DO IF I WANT TO STOP PARTICIPATING IN THIS STUDY?

You can decide to stop participating in this study at any time. If you decide to stop participating, speak with your study physician. If you are randomized to the same-day ART group, you will be able to receive the same ART free of charge at GHESKIO if you choose to stop participating in this study.

# WILL I BE PAID TO TAKE PART IN THIS RESEARCH STUDY?

If you participate in this study, you will receive $US 2.50 per visit to cover the cost of your transportation for every study visit that you attend.

WHAT ABOUT CONFIDENTIALITY?

Efforts will be made to keep your personal information confidential. We cannot guarantee absolute confidentiality. Your personal information may be disclosed if required by law. Any publication of this study will not use your name or identify you personally. Your records may be reviewed by the NIH, and by the Institutional Review Boards at GHESKIO, Partners Healthcare, Cornell University, and Florida International University. Your information may also be reviewed by study monitors, and by study staff.

WHAT ARE THE COSTS TO ME?

All outpatient HIV treatment at GHESKIO is provided free of charge to all patients. For patients who require hospitalization due to HIV or non-HIV-related illnesses, GHESKIO will attempt to find funding to cover the cost of hospitalization, as they do for all GHESKIO patients. This will depend on funds that are available to GHESKIO.

WHAT HAPPENS IF I AM INJURED?

Since you are receiving the same HIV medications that non-research patients receive, we do not expect any research-associated injuries from participation in this study. Injuries sometimes happen in research, even when no one is at fault. If you are injured as a result of being in this study, you will be given immediate treatment for your injuries. There are no plans to pay you or give you other compensation for an injury, should one occur. However, you will not be giving up any of your legal rights by signing this consent form.

WHAT ARE MY RIGHTS AS A RESEARCH PARTICIPANT?

Taking part in this study is completely voluntary. You may choose not to take part in this study or leave this study at any time. Your decision will not affect medical care you receive at this site.

We will tell you about new information from this or other studies that may affect your health, welfare, or willingness to stay in this study. If you want the results of the study, let the study staff know.

WHAT DO I DO IF I HAVE QUESTIONS OR PROBLEMS?

For questions or problems related to the study, you may call:

Dr. Jean William Pape: Director of GHESKIO and leading the study

Phone: 2-222-0031/2-222-2241

Dr. Patrice Severe: Site Leader of the Clinical Trials Unit

Phone: 2-222-0031/2-222-2241

Or you may request to speak to them in person at the GHESKIO Centers: #31, Blvd. Harry Truman, Bicentenary, Port-au-Prince.

For questions about your rights as a participant in the study, you may call:

Dr. Rose-Irène Verdier, who is the person in charge of having the rights of the participants in the study respected

Phone: 2-222-0031/2-222-2241

Dr. Alix Lassegue, who is in charge of ethics at the GHESKIO Centers

Phone: 2-222-4743.

SIGNATURE PAGE

If you have read this consent form (or had it explained to you), all your questions have been answered, and you agree to take part in this study, please sign your name below.

____________________________ ___________________________________

Participant’s Name (print) Participant’s Signature and Date

____________________________ ___________________________________

Study Staff Conducting (print) Study Staff’s Signature and Date

_____________________________ ___________________________________

Name of Witness (print) Witness’s Signature and Date

**Permission to Use Medical Information for Patients who Decline Study Participation**

I decline permission to participate in this study. However, I am willing to explain my reason for declining participation.

Reason for Declining Participation: ________________________________________________ ____________________________________________________________________________________________________________________________________________________________________________________________________________________________________________________________________________________________________________________________________________________________________________________________

Also, I give permission for the study staff to review my medical information and include it in study analyses. I understand that my name and address will not be included on any study documents, and that my information will only be identified through a study number.

SIGNATURE PAGE

I decline participation in this study, but permit you to use my medical information, and my reason for declining participation.

____________________________ ___________________________________

Participant’s Name (print) Participant’s Signature and Date

____________________________ ___________________________________

Study Staff Conducting (print) Study Staff’s Signature and Date

_____________________________ ___________________________________

Name of Witness (print) Witness’s Signature and Date
