# Supplementary material for: Same-day HIV testing with initiation of antiretroviral therapy versus standard care for persons living with HIV: A randomized unblinded trial
Source: PLoS Med. 2017 Jul 25;14(7):e1002357. doi: 10.1371/journal.pmed.1002357 (PMC5526526; doi:10.1371/journal.pmed.1002357)
Supplement: S3 Text — (PDF) [file pmed.1002357.s003.pdf]

HIV Medication Readiness Scale*

Directions: Read each sentence and place a check mark (√) in front of the response that best corresponds to the way the patient feels.

Choices:

0 = not at all ready (patient refuses to do so)
1 = somewhat not ready (patient does not think s/he can do so)
2 = unsure (patient needs more information before doing so)
3 = somewhat ready (patient accepts s/he has to do so)
4 = completely ready (enthusiastic; agrees completely to do so)

If you were to start taking pills today to treat your HIV, how ready would you be to?

1. Take the medicines for a long time (for example, for several years)?
___ 0 not at all ready
___ 1 somewhat not ready
___ 2 unsure
___ 3 somewhat ready
___ 4 completely ready

2. Carry the medicines with you to all your activities, if you are not going to be at home when it's time for you to take the pills?
___ 0 not at all ready
___ 1 somewhat not ready
___ 2 unsure
___ 3 somewhat ready
___ 4 completely ready

3. Take the pills each day as prescribed, as close to the same time as possible?
___ 0 not at all ready
___ 1 somewhat not ready
___ 2 unsure
___ 3 somewhat ready
___ 4 somewhat ready

4. Change your lifestyle in such a way to make sure that you take your medicines as close as possible to the specified time every day?
___ 0 not at all ready
___ 1 somewhat not ready
___ 2 unsure
___ 3 somewhat ready
___ 4 somewhat ready

5. Wear a watch or carry a cell phone to remind you to take your pills?
___ 0 not at all ready
___ 1 somewhat not ready
___ 2 unsure
___ 3 somewhat ready
___ 4 completely ready

6. Go to bed and wake up as close as possible to the same time so as not to forget to take your pills on time?
___ 0 not at all ready
___ 1 somewhat not ready
___ 2 unsure
___ 3 somewhat ready
___ 4 completely ready

7. Continue taking your pills even if you don't feel well (for example, you have diarrhea, you are vomiting, you gain weight)?
___ 0 not at all ready
___ 1 somewhat not ready
___ 2 unsure
___ 3 somewhat ready
___ 4 completely ready

*_Adapted from Balfour L et al. Development and validation of the HIV Medication Readiness Scale. Assessment. 2007 Dec; 14(4): 408-16._

Echèl pou wè si yon pasyan pare pou kòmanse tretman*

Enstriksyon : Tanpri li fraz la epi fè yon tchèk (√) devan repons ki pi byen koresponn ak jan pasyan a santi l.

Repons:
0 = non, pa pare ditou (li refize fè sa)
1 = yon jan pa pare (li pa panse li ka fè sa)
2 = mwen pa konnen (li bezwen plis enfòmasyon pou li ka fè sa)
3 = yon jan pare (li aksepte li gen pou fè sa)
4 = wi, toutafè pare (antouzyas ; san pou san dakò li ka fè sa)

Si w tap kòmanse pran medikaman ki pou trete VIH/SIDA jodi a, èske wap pare pou:

1. Pran medikaman an pou byen lontan (pa egzanp, plizyè ane)?
___ 0 non, pa pare ditou
___ 1 non, yon jan pa pare
___ 2 mwen pa konnen
___ 3 wi, yon jan pare
___ 4 wi, toutafè pare

2.   Èske wap pare pou pote medikaman w nan tout aktivite w si ou pap lakay ou lè pou bwè grenn yo.
___ 0 non, pa pare ditou
___ 1 non, yon jan pa pare
___ 2 mwen pa konnen
___ 3 wi, yon jan pare
___ 4 wi, toutafè pare

3. Èske wap pare pou bwè grenn medikaman yo jan yo di w pou pran yo, toujou apepre menm lè a?
___ 0 non, pa pare ditou
___ 1 non, yon jan pa pare
___ 2 mwen pa konnen
___ 3 wi, yon jan pare
___ 4 wi, toutafè pare

4. Èske wap pare pou viv lavi w yon jan kote w kapab pwomèt wap pran medikaman an menm lè chak jou oswa apepre menm lè chak jou?
___ 0 non, pa pare ditou
___ 1 non, yon jan pa pare
___ 2 mwen pa konnen
___ 3 wi, yon jan pare
___ 4 wi, toutafè pare

5. Èske wap pare pou pote yon mont oubyen yon selilè ki pou fè w sonje lè pou pran medikaman w?
___ 0 non, pa pare ditou
___ 1 non, yon jan pa pare
___ 2 mwen pa konnen
___ 3 wi, yon jan pare
___ 4 wi, toutafè pare

6. Èske wap pare pou dòmi epi leve apepre menm lè chak jou pou w pa bliye pran medikaman w a lè?
___ 0 non, pa pare ditou
___ 1 non, yon jan pa pare
___ 2 mwen pa konnen
___ 3 wi, yon jan pare
___ 4 wi, toutafè pare

7. Èske wap pare pou kontinye pran medikaman w menm si w pa santi w byen (pa egzanp, si w gen vomi, dyare, oubyen w vin pi gwo)?
___ 0 non, pa pare ditou
___ 1 non, yon jan pa pare
___ 2 mwen pa konnen
___ 3 wi, yon jan pare
___ 4 wi, toutafè pare

*Adapted from Balfour L et al. Development and validation of the HIV Medication Readiness Scale. <u>Assessment.</u> 2007 Dec; 14(4): 408-16 and translated in Haitian Creole
